# Genotype by environment interaction for gene expression in *Drosophila melanogaster*

**DOI:** 10.1038/s41467-020-19131-y

**Published:** 2020-10-28

**Authors:** Wen Huang, Mary Anna Carbone, Richard F. Lyman, Robert R. H. Anholt, Trudy F. C. Mackay

**Affiliations:** 1 Program in Genetics, Department of Biological Sciences, W. M. Keck Center for Behavioral Biology, North Carolina State University, Raleigh, NC 27695-7614 USA; 2 Department of Animal Science, Michigan State University, East Lansing, MI 48824 USA; 3 Clemson Center for Human Genetics, Clemson University, Greenwood, SC 29646 USA; 4 Present Address: Center for Integrated Fungal Research and Department of Plant and Microbial Biology, North Carolina State University, Raleigh, NC 27695-7244 USA

**Keywords:** Evolutionary genetics, Gene expression, Genomics, Quantitative trait

## Abstract

The genetics of phenotypic responses to changing environments remains elusive. Using whole-genome quantitative gene expression as a model, here we study how the genetic architecture of regulatory variation in gene expression changed in a population of fully sequenced inbred *Drosophila melanogaster* strains when flies developed in different environments (25 °C and 18 °C). We find a substantial fraction of the transcriptome exhibited genotype by environment interaction, implicating environmentally plastic genetic architecture of gene expression. Genetic variance in expression increases at 18 °C relative to 25 °C for most genes that have a change in genetic variance. Although the majority of expression quantitative trait loci (eQTLs) for the gene expression traits in the two environments are shared and have similar effects, analysis of the environment-specific eQTLs reveals enrichment of binding sites for two transcription factors. Finally, although genotype by environment interaction in gene expression could potentially disrupt genetic networks, the co-expression networks are highly conserved across environments. Genes with higher network connectivity are under stronger stabilizing selection, suggesting that stabilizing selection on expression plays an important role in promoting network robustness.

## Abstract (web summary)

Huang et al. show that developing under different temperatures changes the genetic architecture of regulatory variation in Drosophila melanogaster gene expression yet the co-expression network remains robust. Data suggest that stabilizing selection on gene expression may promote co-expression network robustness.

## Introduction

Organisms living in fluctuating environments or entering novel environments must possess mechanisms to cope with environmental changes. One such mechanism is to change expressed phenotypes in response to different environments, a phenomenon called phenotypic plasticity^1^. Alternatively, organisms may develop homeostatic mechanisms to cushion the effect of environmental fluctuations without changing their phenotypes. The maintenance of homeostasis is important for organismal fitness as it protects organisms from detrimental effects. The definition of environment can be broad, ranging from cell types, tissues, and physiological states, to diseases, external stimuli, and climate; all of which are known to cause plastic changes or lack thereof in certain phenotypes.

In addition to environmental factors, phenotypes can also respond to genetic perturbations in a plastic or homeostatic manner, which characterizes the potential of an organism to express phenotypes when genes mutate. In a population of genetically diverse individuals, the extent of genetic variation of a phenotype measures the overall sensitivity of individuals to mutations segregating in the population.

Importantly, the state of plasticity or homeostasis, with respect to either genetic or environmental variation, is not necessarily static and can be modified by both genetic and environmental factors. A classic example is the heat shock protein system, particularly *Hsp90*, whose expression is environmentally plastic and increases under thermal stress, but buffers phenotypic changes induced by mutations to maintain homeostasis^2^, a process termed canalization^3^. The opposite of canalization—decanalization—describes the change from a homeostatic state to a plastic one, which allows phenotypic expression of genetic and/or environmental variation^4,5^. The dynamics of genetic variation (variance across different genotypes) and environmental variation (variance across different environments) may be controlled by different mechanisms. For example, although the histone variant H2A.Z is a capacitator for environmental variation^6^, its presence in the yeast genome does not increase robustness to mutations^7^.

Change in genetic variation across environments is one of the many forms of genotype by environment interaction (G×E). G×E can be interpreted equivalently either as variable genetic architecture across environments or as variable environmental plasticity across genotypes, depending on what factor is chosen as the context. G×E has important implications in quantitative trait variation and evolution. It is important for maintenance of genetic variation^8^. It is pervasive in plants and animals and influences domestication^9^ and genetic improvement^10,11^. G×E is also of paramount importance to realize personalized medicine such as individualized drug therapy^12^.

Gene expression is a unique class of quantitative traits that are under genetic control and that exhibit both plasticity and homeostasis^13^. Because of the sheer number of gene expression traits and their biological function annotations, gene expression can serve as an important model for quantitative traits.

In this study, we sought to understand G×E for gene expression by exposing the sequenced inbred lines of the *Drosophila melanogaster* Genetic Reference Panel (DGRP) to a low-temperature treatment. Temperature is one of the most wide ranging environmental factors an organism can experience and must manage, and previous studies have shown that there is genetic variation in plasticity of fitness in response to low temperature in *Drosophila melanogaster*^14^. We measured whole-genome gene expression and combined it with a previous data set that quantified gene expression of the same lines reared at standard ambient temperature^15^. This experimental design whereby the same standing genetic variation is subjected to different thermal environments enabled us to address three fundamental questions. First, how does genetic variance of gene expression change when the environment changes? Second, is environmental plasticity of gene expression heritable; or equivalently, does heritable regulatory variation change in response to environmental change? If so, what are the locations and effects of the quantitative trait loci (QTLs) that exhibited such variability and response to environments? And finally, how does the response of regulatory genetic variation in gene expression changes the architecture of co-expression networks?

## Results

### Identification of transcriptional units by RNA sequencing and estimation of gene expression by tiling microarrays

**Fig. 1:**
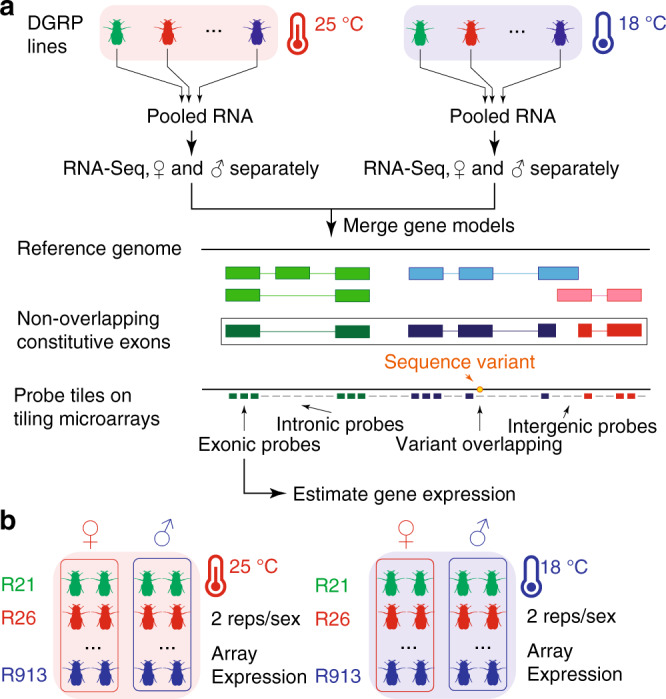
Experimental design and gene expression estimation strategy. **a** RNA extracted from adult flies raised at 25 and 18 °C from all DGRP lines was pooled and sequenced to define a set of nonoverlapping constitutive exons. Genome tiling arrays were then used to estimate gene expression, summarizing expression based on intensities of probes that fall entirely within constitutive exons and do not overlap with common sequence variants. **b** DGRP lines were raised at 25 and 18 °C and RNA was extracted from adult flies for two biological replicates per sex within each temperature. Gene expression was estimated using genome tiling arrays.

We used a two-stage procedure (Fig. 1a) to measure whole-genome gene expression profiles for adult flies independently raised at 18 and 25 °C for 185 DGRP lines^16^. In the first stage, we defined regions in the genome that expressed detectable RNA levels by sequencing pooled polyadenylated RNA from all DGRP lines for each of the two sexes, separately for each of the two temperatures (Supplementary Data 1 and Supplementary Fig. 1). After alignment of RNA sequence reads to the reference transcriptome and genome, followed by transcript model reconstruction, we merged known and newly discovered transcript models from all conditions to obtain 21,873 gene models with nonoverlapping constitutive exons (Fig. 1a).

In the second stage, we used genome tiling arrays to estimate gene expression for the DGRP lines with two replicates per line, sex, and temperature (Fig. 1b). After removing probes that overlapped with common non-reference alleles, we were able to estimate expression for 20691 genes, among which about 24% (*n* = 4943) were unannotated novel transcribed regions (NTRs). Our subsequent global analyses did not differentiate between annotated genes and NTRs, except when performing gene set enrichment analyses when annotations were needed. We filtered out six samples (none involved both replicates of the same condition) that were outliers based on scaled expression within each sex and temperature (Supplementary Fig. 2) and removed undesired batch effects using surrogate variable analysis^17^ on normal quantile-transformed expression within each sex. The adjustment appeared to be effective because known batches such as array scan date was effectively captured by the derived surrogate variables (Supplementary Fig. 3). Subsequent analyses began with these adjusted expression values.

### Canalization and decanalization of genetic variance in gene expression

Because we collected the data at 18 and 25 °C separately, the effects of temperature and batch were confounded. Therefore we cannot make inferences on the effect of temperature on the mean change of gene expression, which has been extensively investigated in other studies^18,19^. To characterize the patterns of genetic variance in gene expression in the two thermal environments, we first partitioned the variance in gene expression into the between-line genetic component ($\sigma _G^2$) and the remaining within-line microenvironmental component ($\sigma _e^2$). In females, there were 4912 genes with significant genetic variance at 18 °C, and 3002 at 25 °C at an false discovery rate (FDR) = 0.05 (Supplementary Data 2), among which 2505 were shared between both environments. In males, there were 5315 and 4278 genes with significant genetic variance at 18 and 25 °C, respectively, including 3339 in common between the two environments (Supplementary Data 2).

**Fig. 2:**
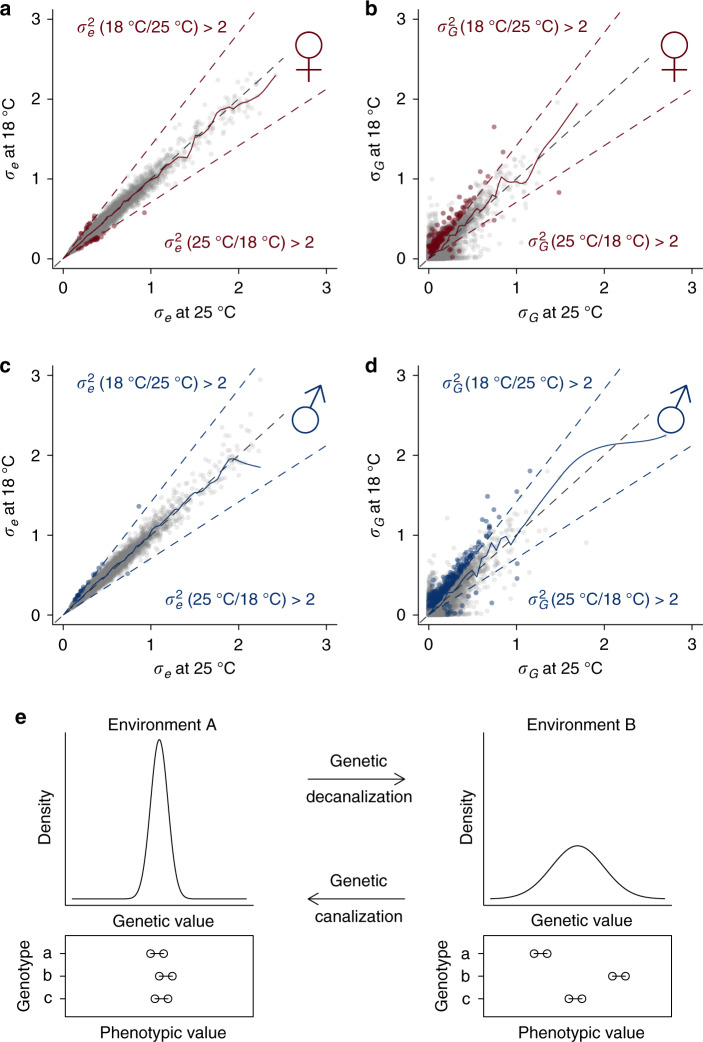
Decanalization and canalization of genetic variance of gene expression at 18 °C. **a** Comparison of environmental variation of gene expression between 18 and 25 °C in females. The two outer dashed lines indicate twofold changes, while the diagonal line indicates no change. Note that the axes are standard deviations while the twofold change are assessed for variances. A LOESS smooth line (dark solid line) is added with a span of 0.001 at the scale of the plotted axis. Red points indicate genes with significant variance heterogeneity for $\sigma _e^2$ (FDR = 0.05) by at least twofold. **b** Comparison of genetic variation of gene expression between 18 and 25 °C in females. The dashed lines indicate twofold changes, while the diagonal line indicates no change. A LOESS smooth line (dark solid line) is added with a span of 0.001 at the scale of the plotted axis. Red points indicate genes with significant variance heterogeneity for $\sigma _G^2$ (FDR = 0.05) by at least twofold and significant $\sigma _G^2$ (FDR = 0.05) in the environment with higher genetic variance. **c**, **d** Comparison of environmental and genetic variances in males, where blue points indicate genes with significant variance heterogeneity. **e** Plots illustrating the dynamics of genetic variances (genetic decanalization/canalization) between two environments. The density plots show the distributions of genetic values for individuals in the same population in two environments (A and B). Below the density plots are line graphs showing phenotypic values where points connected by lines are biological replicates from the same genotypes to illustrate microenvironmental variance.

The marked difference in the numbers of genes with significant genetic variance at 18 and 25 °C in both sexes was intriguing. This could be due to either an overall decrease in environmental variance or an increase in genetic variance at 18 °C. We therefore tested for variance heterogeneity for the genetic and environmental components. In both sexes, $\sigma _e^2$ was relatively stable across the two temperatures for the majority of genes (Fig. 2a–d and Supplementary Data 2). In contrast, the difference in $\sigma _G^2$ was far more pronounced (Fig. 2a–d and Supplementary Data 2). This pattern of variance heterogeneity was almost identical when we did not adjust for infection status with the symbiont *Wolbachia* bacteria that affects approximately half of the lines (Supplementary Fig. 4). This is consistent with the previous observation that *Wolbachia* infection does not substantially impact gene expression^15,20^.

We define genetic decanalization as increased genetic variance in gene expression, and genetic canalization as decreased genetic variance in an environment relative to an arbitrary baseline (Fig. 2e). Our tests for variance heterogeneity at the two temperatures revealed both genetic decanalization and canalization for gene expression relative to 25 °C when flies developed at 18 °C, depending on specific genes considered (Fig. 2). Interestingly, genetic decanalization at 18 °C relative to 25 °C was more prevalent than canalization. There were 149 genes in females that had significantly different genetic variance at 18 than 25 °C by at least twofold. Among these genes 141 were genetically decanalized and only 8 were canalized (Supplementary Data 2). The same was true in males, where 264 genes were decanalized and 15 were canalized at 18 °C. We found little evidence for preferential genetic canalization or decanalization of the expression of genes involved in particular functions. Using gene set enrichment analysis (GSEA), only three broad gene ontology (GO) terms were found to be significantly (FDR = 0.05) enriched for genes whose genetic variance was decanalized or canalized at 18 °C, including the structural constituent of chitin-based cuticle that was enriched for decanalized genes in females and odorant binding and DNA-binding transcription factor activity enriched for canalized genes in males (Supplementary Data 3 and Supplementary Fig. 5).

### Response of regulatory genetic variation in gene expression to environmental change

**Fig. 3:**
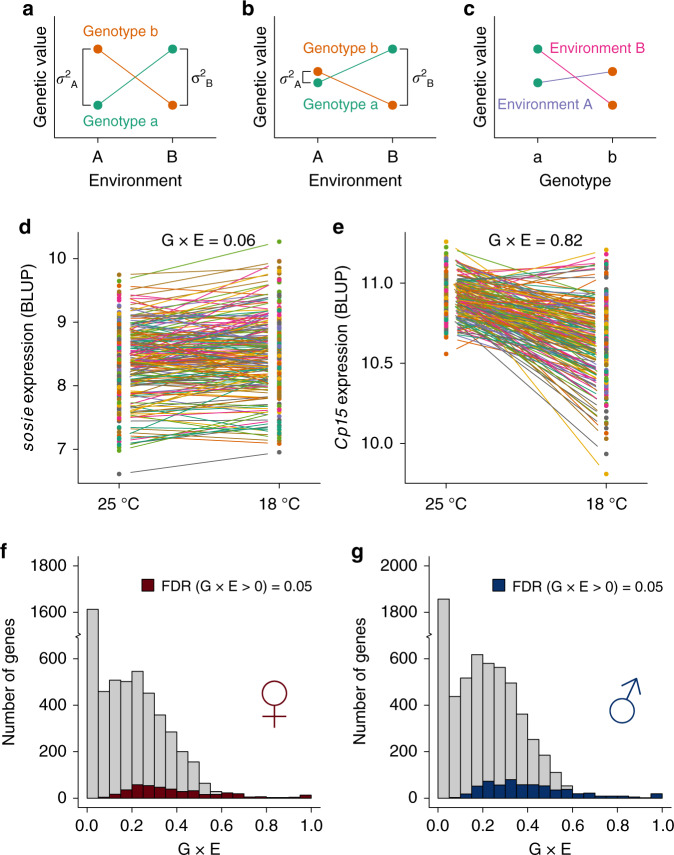
Genotype by environment interaction (G×E) of gene expression. **a** Reaction norms between environment A and B for two genotypes a and b. Points of the same color represent the same genotype; the slope of the lines connecting points of the same color indicate the magnitude of the change in genetic value for that genotype between the two environments. Significant differences in slope for different genotypes signify the presence of G×E. In this example, there is G×E but no change in genetic variance between the two environments. **b** No mean effect of temperature but significant G×E and change in genetic variance between the two environments. **c** The same data as in **b** but plotted with respect to difference between genotypes across the two environments. **d** Example of a gene (*sosie*) in females with low degree of G×E. **e** Example of a gene (*Cp15*) with a high degree of G×E in females. G×E is defined as ${\mathrm{G{\times}E}} = \sigma _{{\mathrm{GE}}}^2/(\sigma _{{\mathrm{GE}}}^2 + \sigma _G^2)$. **f** Distribution of G×E for genes in females, where significant genes are marked by dark red color. **g** Distribution of G×E for genes in males, where significant genes are marked by dark blue color.

The change in genetic variance upon exposure to environmental change is a special form of G×E. To understand the genetic basis of G×E in gene expression, we asked whether there was variation in gene expression that could be attributed to G×E. There are several equivalent ways to describe this phenomenon, each with a different perspective. The first is a largely statistical description, which can be graphically illustrated by reaction norms. In the reaction norm representation, the presence of G×E causes otherwise parallel lines depicting environmental plasticity to cross (Fig. 3a). G×E may or may not be accompanied by an environmental effect when averaged across individuals. Importantly, with the same phenotypic scale across environments, a change in genetic variance will cause a statistical presentation of G×E (Fig. 3b) though the reverse is not necessarily true. Alternatively, G×E is equivalent to environmentally responsive differences between genotypes (Fig. 3c). If environmental plasticity is genetically variable, its variation between DGRP lines characterizes the degree to which the plasticity is heritable (Fig. 3a). Finally, if an environment is able to modify the allelic effects of QTLs controlling the phenotype^21–23^, significant G×E indicates that the genetic architecture of the phenotype is environmentally responsive.

To identify genes that showed significant G×E, we pooled data from both environments and partitioned the variance in gene expression into components due to a common genetic effect shared by both temperatures ($\sigma _G^2$) and due to G×E ($\sigma _{{\mathrm{GE}}}^2$). Remarkably, among the 5248 genes in females and 6327 genes in males that had at least some significant genetic component ($\sigma _G^2$ or $\sigma _{{\mathrm{GE}}}^2$), 424 (8%) and 619 (10%), respectively, had significant G×E at an FDR = 0.05 (Supplementary Data 2 and Fig. 3d, e). The genetic architecture of regulatory variation of these genes was thus variable or environmentally responsive between the two thermal environments. Equivalently, the environmental plasticity of these genes in response to low temperature was therefore heritable. Of these genes, 66 and 110 were also significant for variance heterogeneity between the two environments in females and males, respectively. GSEA revealed little evidence of G×E or the lack of it concentrating in particular biological functions, as only a very limited number of broad GO terms were enriched for higher or lower degrees of G×E. GO terms enriched (FDR = 0.05) for G×E include tricarboxylic acid cycle and translation initiation, which are enriched for greater G×E in males; and protein serine/threonine kinase activity, which is enriched for less G×E in males (Supplementary Data 4).

### Mapping response of regulatory genetic variation to environmental change

We have previously shown that we can map expression quantitative trait loci (eQTLs) for a substantial fraction of genes that are genetically variable in the same population^15^. To understand the environmental response of the genetic architecture of gene expression, we mapped eQTLs at both 18 and 25 °C among 1891697 common (minor allele frequency >0.05) variants and compared the locations and effects of the eQTLs. In females, there were 793 and 511 genes with at least one mapped eQTL (FDR = 0.05), constituting ~16% and 17% of the genetically variable genes, at 18 and 25 °C, respectively. In males, we mapped eQTLs (FDR = 0.05) for 1086 (20%) and 808 (19%) genes at 18 and 25 °C, respectively. To refine the eQTL-gene association models and account for linkage disequilibrium between DNA variants, we performed forward model selection. This procedure sequentially added variants meeting the FDR thresholds in the order of their association with gene expression, conditional on existing variants in the model, until no variant could be added at *P* < 1 × 10^−5^. It resulted in between 1 and 5 eQTLs for each gene, with the majority (74%) of genes containing only one eQTL (Supplementary Data 5).

**Fig. 4:**
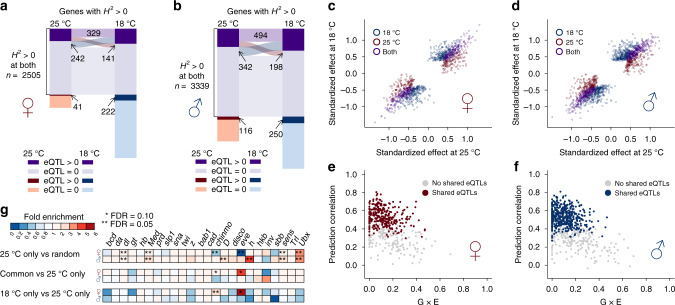
Plasticity of regulatory variation in gene expression. Sharing of genes with eQTLs between 25 and 18 °C in females (**a**) and males (**b**). The numbers indicate genes only. Each colored box represents the number of genes in each category, with the category indicated on the bottom left, where eQTL > 0 indicates genes with mapped eQTLs while eQTL = 0 indicates genes without mapped eQTLs. Boxes with the same colors (dark and light purple) are genes that are genetically variable at both temperatures. The extent of sharing is proportional to the width of the ribbons connecting the boxes. eQTL effect comparison between 25 and 18 °C in females (**c**) and males (**d**). Cross-environment prediction of gene expression using eQTLs in females (**e**) and males (**f**). Gene expression at 18 °C was predicted based on eQTLs mapped at 25 °C and the prediction was evaluated by Pearson correlation between the observed and predicted expression. The prediction correlation is plotted against G×E. **g** Enrichment/depletion of transcription factor binding sites overlap among eQTLs. The enrichment was computed based on the ratio over the mean of overlap among 10,000 randomly drawn sets of eQTLs with the same numbers across the three classes (25 °C only, 18 °C only, and common). The *P* value for enrichment was computed based on the number of iterations that was more extreme (one-sided, enrichment or depletion) than the observed overlap and adjusted using the Benjamini–Hochberg approach.

Using the mapped eQTLs and their estimated effects and locations, we made four comparisons in order to understand the response of the regulatory genetic variation to environmental change in the Drosophila transcriptome. First, we asked if there were shared or environment-specific eQTLs, regardless of the signs and magnitudes of effects. In females, there were 2407 and 497 genes that had environment-specific genetic variance at 18 and 25 °C, respectively, among which 222 (9%) and 41 (8%) contained mapped eQTLs (Fig. 4a). In males, 1976 and 939 genes were genetically variable only at 18 and 25 °C, respectively; 250 (13%) and 116 (12%) of which contained mapped eQTLs (Fig. 4b). These environment-specific eQTLs represent regulatory genetic variation that responds (“inactive” versus “active”) to the specific environmental change. In addition, of the 2505 genetically variable genes at both 18 and 25 °C in females, 571 and 470 genes had mapped eQTLs, respectively (Fig. 4a). Among the 329 genes with at least one eQTL in both environments (sharing of the dark purple boxes in Fig. 4a), 303 (92%) had at least one shared eQTL. A similar result was obtained for males, where 459 (93%) out of 494 genes with eQTLs in both environments shared common eQTLs (Fig. 4b). These results indicate that when genes contained eQTLs in both environments, the majority of them shared eQTLs.

Second, we compared the estimated single-variant effects of eQTLs at 18 and 25 °C among the genes that were genetically variable at both temperatures. Of the 1181 eQTL-gene pairs in females, 294 (25%) were specific to 25 °C, 436 (37%) were specific to 18 °C, and 451 (38%) were common to both (Fig. 4c). Of the 1740 eQTL-gene pairs in males, 412 (24%) were specific to 25 °C, 630 (36%) were specific to 18 °C, and 698 (40%) were common to both (Fig. 4d). However, these classifications depended on significance thresholds. To enable a more quantitative comparison, we estimated single-variant genetic effects regardless of their statistical significance as long as it was significant in at least one environment. As expected, for eQTLs that were shared by both environments, their effects were large and highly similar (Fig. 4c, d). On the other hand, eQTLs that were specific to either environment had much larger effects in the environment where they were detected (Fig. 4c, d). Those eQTLs whose effects were different between environments also contributed to the environmental response of the regulatory variation. Interestingly, almost all eQTLs had effects of the same sign in both environments, suggesting that G×E or the environmental response of regulatory genetic variation for the majority of genes was likely a result of change in the magnitudes of effects rather than signs.

Third, we asked if using the estimated eQTL effects in one environment could predict gene expression in the other. Conserved regulatory genetic variation would lead to better prediction accuracy. We predicted gene expression at 18 °C for each gene using mapped eQTLs and their effects at 25 °C. As expected, the prediction accuracy correlated well with G×E. Genes with higher G×E, which also tended to not share eQTLs in the two environments, were poorly predicted across environments (Fig. 4e, f).

Finally, to probe the nature of the regulatory genetic effects on plasticity, we compared the degree of overlap between the eQTLs of different classes (shared or environment-specific) with known transcription factor binding sites in the Drosophila genome. In both females and males, eQTLs tended to overlap with transcription factor binding sites in general (Fig. 4g, 25 °C only vs. random), consistent with their localization to the proximity of transcription start and end sites^15^. Two developmentally important transcription factors, *chinmo* and *eve*, were significantly (FDR = 0.05) depleted among eQTLs specific to 25 °C in females (Fig. 4g). This led to the over-representation of eQTLs common to both environments (FDR = 0.10) or specific to 18 °C (FDR = 0.05 for *chinmo* and 0.10 for *eve*) overlapping the binding sites of these two transcription factors (Fig. 4g). Therefore, *chinmo* and* eve* may be involved in the environment-specific regulation of gene expression in females.

### Robust co-expression networks in the presence of environmentally responsive regulatory genetic variation

It has been well documented that genes form co-expression networks in which genes executing similar functions have correlated expression levels, either across different tissues of the same individuals, or across individuals in a population^24^. Importantly, perturbations to co-expression networks can lead to expressed phenotypes such as diseases^25,26^. In a population sample, if there was no genetic variation in the plasticity of gene expression, the co-expression networks constructed based on correlations between genes would remain the same even if plasticity was widespread but constant across individuals. In contrast, G×E or plasticity of regulatory variation may lead to a change in the network structure.

**Fig. 5:**
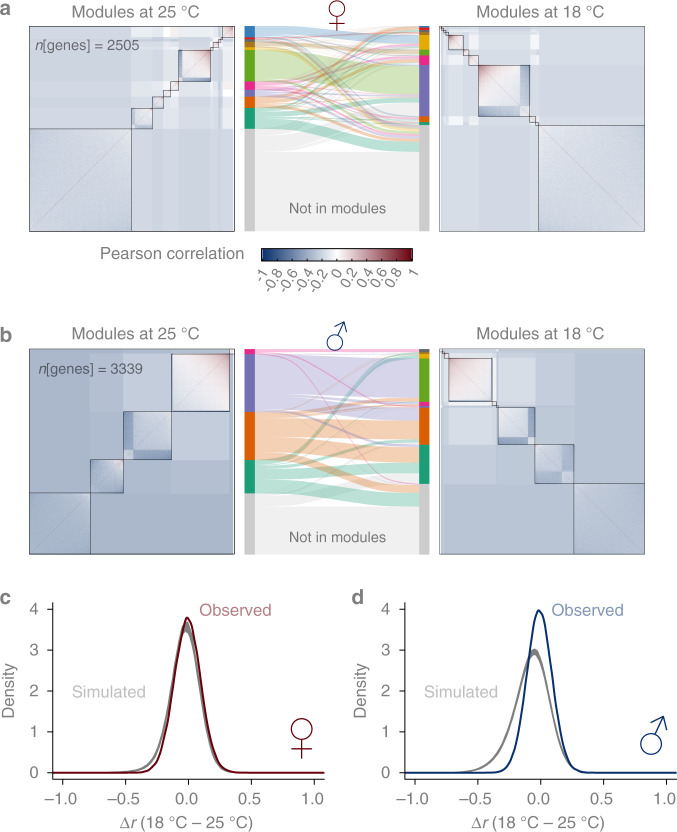
Robust co-expression networks in the presence of plastic regulatory variation. Module preservation between 25 and 18 °C in females (**a**) and males (**b**). Density plots for ∆*r* between 18 and 25 °C where the gray lines are based on 1000 simulations while the darkcolored line is observed in females (**c**) and males (**d**). The test for significance was based on the variance of the distribution of ∆*r*. The null hypothesis was equal variance while the alternative hypothesis was smaller variance.

To examine the robustness of co-expression networks in the presence of heritable transcriptome plasticity, we identified co-expression modules using weighted correlation network analysis (WGCNA) in both temperatures and compared the module memberships (Fig. 5a, b). We observed strong preservation of network structures. For example, in females, the largest module at 25 °C included 386 genes enriched for basic biological processes such as neurogenesis, mitotic spindle organization, and translation, among others (Supplementary Data 6). Of these genes, 90% (*n* = 346) were also in the same module at 18 °C (Fig. 5a). Using a permutation-based approach^27^, several measures of network preservation were found to be highly significant (Supplementary Data 7).

However, given that only ~10% of genes showed G×E or plasticity of regulatory variation, a high degree of preservation was expected, especially for genes that are required to maintain basic organismal functions. To test the trivial explanation that the network structure preservation is expected given the observed extent of G×E in the transcriptome, we employed a simulation-based approach to derive the expected distribution of changes in correlation under the null hypothesis that the plastic changes in the regulatory variation of genes were independent from each other (Fig. 5c, d). We found evidence that the changes in the correlation among genes were smaller than if all genes were responding to the environment change independently. Between the genes with significant G×E, the change in their correlation with each other was significantly smaller than expected (*P* < 0.001 based on 1000 simulations), more so in males than in females (Fig. 5c, d). This result indicates that mechanisms exist to ensure that plasticity of regulatory variation in gene expression remains coordinated between genes in the face of environmental perturbations, thus preserving the co-expression networks.

**Fig. 6:**
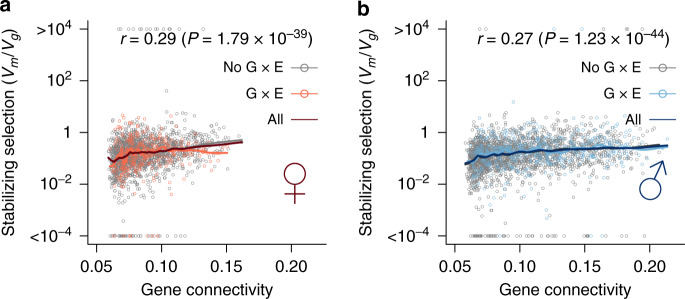
Positive correlation between gene connectivity and strength of stabilizing selection. The strength of stabilizing selection as measured by the ratio between mutational variance (*V*_*m*_) and standing genetic variance (*V*_*g*_) is plotted against gene connectivity (mean |*r*| for expression at 25 °C) in females (**a**) and males (**b**). The dark lines are LOESS smooth lines with a span of 0.1. The colors of the points and lines indicate genes with no G×E, G×E, or all genes. Spearman’s correlation (*r*) and the corresponding *P* values were also indicated for all genes. In females (**a**), the Spearman’s correlation (*P* value) is 0.30 (*P* = 1.06 × 10^−37^) for genes without G×E, 0.19 (*P* = 5.11 × 10^−4^) for genes with G×E. In males (**b**), the Spearman’s correlation (*P* value) is 0.27 (*P* = 5.06 × 10^−35^) for genes without G×E, 0.21 (*P* = 3.89 × 10^−6^) for genes with G×E.

If the environmental response of regulatory genetic variation is not independent but coordinated, genes that are highly correlated with a large number of genes (“hub” genes) may be of particular importance in preserving the co-expression networks. We postulated that expression of these genes may be under stronger constraints. We tested this hypothesis by relating the connectivity of genes with the strength of stabilizing selection estimated in a previous study^28^. We defined the connectivity of a gene as the mean of the absolute correlation of the gene with all other genes, and the strength of stabilizing selection as the ratio of mutational variance (*V*_*m*_) over standing genetic variation (*V*_*g*_). Remarkably, there was a highly significant positive correlation (Spearman *r* = 0.29 and *P* = 1.79 × 10^−39^ in females and *r* = 0.27 and *P* = 1.23 × 10^−44^ in males) between a gene’s network connectivity and the strength of stabilizing selection in both sexes (Fig. 6a, b), indicating that the robustness of co-expression networks is under stabilizing selection. The correlation was slightly stronger for genes without G×E (Fig. 6a, b). Though conceivable and suspected in many cases, few studies have experimentally observed this result.

## Discussion

Using a simple but powerful design, we provided a comprehensive characterization of the response of the regulatory genetic variation of the Drosophila transcriptome to environmental change. Specifically, an inbred line reference population enabled us to use the same genotypes for the alternative treatments and obtain biological replicates. This was instrumental in partitioning the variance in gene expression and providing a global characterization of the extent of genetic canalization or decanalization and G×E. Although we found many more genes genetically decanalized at 18 °C relative to the baseline at 25 °C, this depended on the choice of the baseline environment as well as the environmental history of this population that had shaped its genetic variation. Nevertheless, the genetic variance of hundreds of genes changed between the two thermal environments, clearly indicating that even mild environmental fluctuations can be a potent agent in exposing or masking genetic variation in quantitative trait phenotypes. In addition, ~8–10% of genetically variable genes exhibited G×E in just two environments. These genes may be subject to differential selection when they experience heterogeneous environments, contributing to the maintenance of genetic variation^8^. Because only two environments were considered in this study, the extent of G×E may be much more widespread than can be detected and all inferences drawn from this study should be interpreted in the specific context of the two temperatures.

We compared the list of genes exhibiting G×E in the present study with genes that showed evidence of adaptive divergence or G×E between tropical and temperate geographical locations. Among the 619 genes significant for G×E in males in this study, 166 (Supplementary Data 8) were previously reported to be differentially expressed between flies of tropical and temperate origins^29,30^ or had G×E interaction^18^. However, more formal integration across studies with proper experimental design and statistical inference is needed to understand the role of canalization and decanalization and G×E in general in adaptive evolution, which remains controversial^31^.

We mapped eQTLs at 18 and 25 °C and compared their locations and effects. While there were many environment-specific or plasticity eQTLs, when eQTLs could be mapped in both environments, the majority of eQTLs were in fact shared between the two environments with similar effects (Fig. 4a–d). Cross-environment prediction of gene expression using mapped eQTLs was poor in the presence of G×E, a caveat that must be considered when predicting gene expression based on mapped eQTLs in reference populations^32^. We found two transcription factors (chinmo and eve) whose binding sites were enriched in eQTLs in 18 °C relative to 25 °C specific eQTLs, suggesting that they may be involved in regulating the plasticity of gene expression when flies were exposed to 18 °C. The specific mechanisms by which these cis regulatory elements change how flies respond to different temperatures require further investigation. Differential binding by the developmentally dynamic chinmo and eve transcription factors may modify the development of sensory neurons such that flies sense environmental temperature differently^33^.

We observed strong preservation of the co-expression network structure between the two environments. A trivial explanation for this observation could be that since the majority of genes did not show significant G×E, the correlation among these genes was preserved. We developed a simulation-based test to specifically test whether the robustness of co-expression networks was expected under the null hypothesis that the plastic responses to low temperature was independent for each of the significant genes. This hypothesis was rejected, indicating that the plastic responses by the genes were somewhat coordinated. This would be consistent with a model where the responses of many genes were secondary to a smaller number of primary first responders. Our identification of two transcription factors in females whose binding sites were enriched in eQTLs in 18 °C (common between 25 and 18 °C or specific to 18 °C) was also consistent with this model. A robust co-expression network involving many genes would also promote polygenicity of organismal traits or fitness if we consider the expression of genes as mediators of mutational effects on organismal traits. This would be consistent with the ominigenic model of complex quantitative traits^34^. Indeed, adaptation to novel thermal environments has been found to be highly polygenic^35^. However, further experiments are needed to define a working model.

Importantly, we found genes with higher network connectivity with other genes, which were also under stronger stabilizing selection (Fig. 6). Furthermore, larger and more conserved modules were enriched for genes involved in basic biological processes (Fig. 5a, b and Supplementary Data 6). Previous theoretical work has suggested that stabilizing selection was important in promoting the evolution of network robustness^36^. Our result is one of the few^37^ that provides empirical support for the role of stabilizing selection in evolving network robustness.

## Methods

### Sample preparation

For the low-temperature treatment, all DGRP strains were reared on cornmeal-molasses-agar medium at 18 °C with 60–75% relative humidity and a 12-h light/dark cycle. For each DGRP line, we collected 25 mated female or 40 mated male 3–5-day-old flies to constitute one biological replicate for each sex. Collection was performed between 1 and 3 p.m. consistently to account for circadian rhythm in gene expression and the flies were immediately frozen in liquid nitrogen before they were sorted. We collected two biological replicates per sex for each of 185 DGRP lines.

### RNA sequencing and analysis

We sequenced pooled polyadenylated RNA of flies reared at 18 °C exactly as previously described^15^. RNA-Seq data for flies reared at 25 °C were downloaded from GEO (GSE67505) and data from the present study were deposited to SRA (PRJNA615927). Although all analyses were identical, we re-analyzed the 25 °C data together with the new 18 °C data for consistency and improvement in sensitivity by merging transcript reconstructions across environments. Briefly, RNA-Seq reads were aligned to the reference transcriptome (FlyBase release 5.57) and the genome (BDGP5) using tophat2^38^ (v2.0.13) for each sex and temperature separately. A summary of alignments including statistics of input, successful alignments, and reads overlapping various genomic features is provided in Table S1. Alignments were assembled using cufflinks^39^ (v2.2.1) into transcript models, which were merged across multiple conditions. To enable subsequent expression profiling using microarrays, we removed segments of exons that were either specific to only a subset of splice isoforms or overlapped from either strand by multiple genes to arrive at a set of nonoverlapping constitutive exons for each gene (Fig. 1).

### Microarray data acquisition and processing

We acquired and processed microarray data as previously described^15^. The 25 °C data were downloaded from ArrayExpress (E-MTAB-3216) and analyzed together with the new 18 °C data (E-MTAB-8953). We removed probes that mapped to multiple genomic locations, overlapped with variants whose non-reference allele frequency in the 185 DGRP lines exceeded 0.05, or did not entirely fall within the constitutive exons as defined above. Probe hybridization intensity was corrected for background hybridization^40^ and quantile normalized^41^ within each sex but across temperature, which was motivated by strong effects of sex on gene expression^15^ but relatively minor temperature effects as observed in the RNA-Seq data (Supplementary Fig. 1), and an attempt to partially account for batch effects. Probe expression was summarized into gene expression by median polish, which is robust to rare outliers. Finally, within each sex, the expression for each gene was normal quantile-transformed with mean equal to the median and standard deviation equal to the median absolute deviation multiplied by a factor of 1.4824. It is important to note that because the 18 and 25 °C data were collected at different times, batch is completely confounded with temperature, and therefore we could not make any inference about the overall effect of temperature on gene expression. Nonetheless, because the arrays were randomized within each temperature, we could still make inferences on G×E after the temperature effect and other latent batch effects were accounted for. We removed unwanted heterogeneity in the gene expression matrix by regressing out top surrogate variables while explicitly retaining temperature and *Wolbachia* effects^17^. The numbers of surrogate variables were determined using the “num.sv” function in the “sva” package (v3.34) in R. The adjusted gene expression matrices were used for subsequent analyses for females and males separately.

### Quantitative genetic analysis of gene expression

Within each temperature and sex, we partitioned the observed phenotypic variance into genetic ($\sigma _G^2$) and microenvironmental ($\sigma _e^2$) variances using mixed model implemented in the “lme4” R package (v1.1–10), adding *Wolbachia* infection status as a fixed effect. We then combined data from the two temperatures and partitioned the observed phenotypic variance into genetic and environmental components using different models as described below, which allowed us to make specific inferences. First, we tested variance homogeneity by likelihood ratio tests comparing models with either a single variance parameter or separate variance parameters for the environments. The models were fitted by the “nlme” R package (v3.1–124) and included temperature, *Wolbachia*, and the interaction between the two as fixed effects. Second, we also partitioned the observed phenotypic variance across environments into genetic ($\sigma _G^2$), G×E interaction ($\sigma _{{\mathrm{GE}}}^2$), and microenvironmental variances ($\sigma _e^2$), and the same fixed effects as above. We defined a G×E index as ${\mathrm{G{\times}E}} = \sigma _{{\mathrm{GE}}}^2/(\sigma _{{\mathrm{GE}}}^2 + \sigma _G^2)$, which measured the proportion of total genetic variance due to G×E interaction. The FDR was controlled using the Benjamini–Hochberg procedure when calling statistical significance. We compared genes significant for G×E with genes that showed evidence of adaptive divergence or G×E between tropical and temperate geographical locations. These included genes that were differentially expressed between African and European populations^29^; genes that were differentially expressed between Panama City and Maine populations at 21 and 29 °C^30^; and genes that showed G×E from temperate and tropical Australia populations raised at 18 and 30 °C^18^.

### Gene set enrichment analysis (GSEA)

We performed two different quantitative GSEA analyses. The first was a measure of canalization and decanalization and was computed as $(\sigma _{G,\,18\,^\circ {\mathrm{C}}}^2 - \sigma _{G,\,25\,^\circ {\mathrm{C}}}^2)/(\sigma _{G,\,18\,^\circ {\mathrm{C}}}^2 + \sigma _{G,\,25\,^\circ {\mathrm{C}}}^2)$. We considered only genes with at least one significant genetic variance at 18 or 25 °C. The second was a measure of G×E defined as 2G×E − 1 for genes with significant $\sigma _{{\mathrm{GE}}}^2$ and/or $\sigma _G^2$. These scores were designed to range between −1 and 1 and replaced the correlation scores in GSEA, the general form of which takes a ranked list of scores as input. Subsequent steps were exactly followed as previously described in the original GSEA publication^42^. We limited analyses to GO terms with at least 20 genes among the entire set of genes with at least one GO annotation. For GO enrichment analysis for genes in Module 1 of the female WGCNA (v1.69) modules, the significance was tested using a hypergeometric test with genes in the WGCNA input as the background set. *P* values were adjusted for multiple testing using the Benjamini–Hochberg procedure.

### eQTL mapping

We mapped eQTLs as previously described^15^. Briefly, we obtained BLUP estimates of gene expression for each line at 18 °C and 25 °C separately, adjusting for *Wolbachia* infection, inversions, and major genotypic principal components. eQTLs were mapped using a *t* test implemented in PLINK (v1.07). To estimate the empirical FDR, the phenotypic data were permuted 100 times, retaining the association between genes, and eQTLs were mapped following the same procedure. At each *P* value threshold, the empirical FDR was estimated as the average expected number of significant eQTLs divided by the observed number. Significant eQTLs were further filtered by performing a forward selection procedure as previously described^15^. In each iteration of the forward selection, all candidate eQTLs were tested conditional on eQTLs in the model, the one with the smallest *P* value was added to the model until no eQTL could be added at *P* < 10^−5^. When considering matching of eQTLs between environments, an eQTL must be retained in the model selection in at least one environment. For example, an eQTL retained in the model selection at 18 °C may not be retained at 25 °C, but overlap is still assessed as long as it has been initially mapped at 25 °C before model selection.

### Co-expression network analysis

We identified modules in gene expression using the WGCNA (v1.69) R package^43^ and tested network preservation naively using the NetRep (v1.2.1) R package^27^. To account for known cross-environment correlation of gene expression and quantitatively compare the preservation of correlation of expression among genes, we employed a simulation-based approach. To test the null hypothesis that the plastic response of gene expression at 18 °C of different genes were independent from each other, we simulated for each gene its expression at 18 °C given the correlation between 18 and 25 °C. This was achieved by first scaling the expression at 25 °C such that $Z_1 = (X - \mu _1)/\sigma _1$, where *X* was the expression at 25 °C. Then the expression at 18 °C was simulated by the following formula: $Y = \sigma _2(\rho Z_1 + \sqrt {1 - \rho ^2} Z_2){\mathrm{ + }}\mu _2$, where *μ*_2_ and *σ*_2_ were the mean and variance of expression at 18 °C, *ρ* was the correlation coefficient between the expression at the two environments and *Z*_2_ was a random number drawn from the standard normal distribution. Thus *Y* was a simulated gene expression that preserved the mean and variance at 18 °C as well as the correlation and G×E between the two environments. We then computed the pair-wise correlation matrix at both temperatures and computed their difference. This procedure was repeated 1000 times. A significant deviation from the null hypothesis was signified by decreased variance of the distribution of the correlation coefficient changes (Δ*r*).

We defined gene connectivity of a gene as the mean absolute correlation of the gene with all other genes. The strength of stabilizing selection was obtained from a previous study^28^, in which mutational variance (*V*_*m*_) of gene expression was estimated in a set of mutational accumulation lines while the standing genetic variation (*V*_*g*_) was estimated from a subset of DGRP lines. The study also used whole bodies of flies of the same age but a different array platform and thus was an independent study from the present one. The strength of stabilizing selection was defined as the ratio of *V*_*m*_ over *V*_*g*_.

### Reporting summary

Further information on research design is available in the Nature Research Reporting Summary linked to this article.

## Supplementary information

Supplementary Information

Peer Review

Reporting Summary

Description of Additional Supplementary Files

Supplementary Data 1-8

## Data Availability

All data have been deposited into public repositories, including the RNA-Seq data (GSE67505 in GEO for 25 °C, PRJNA615927 in SRA for 18 °C), and the tiling microarray data in ArrayExpress (E-MTAB-3216 and E-MTAB-8953). All derivative data used to generate figures and tables are available in the GitHub repository https://github.com/qgg-lab/dgrp-plasticity-eqtl.

## Code availability

All codes used for analysis and figure and table generations are available in the GitHub repository https://github.com/qgg-lab/dgrp-plasticity-eqtl.
