# Supplementary material for: Genotype by environment interaction for gene expression in Drosophila melanogaster
Source: Nat Commun. 2020 Oct 28;11:5451. doi: 10.1038/s41467-020-19131-y (PMC7595129; doi:10.1038/s41467-020-19131-y)
Supplement: Supplementary file 1 — Supplementary Information [file 41467_2020_19131_MOESM1_ESM.pdf]

Supplementary Information

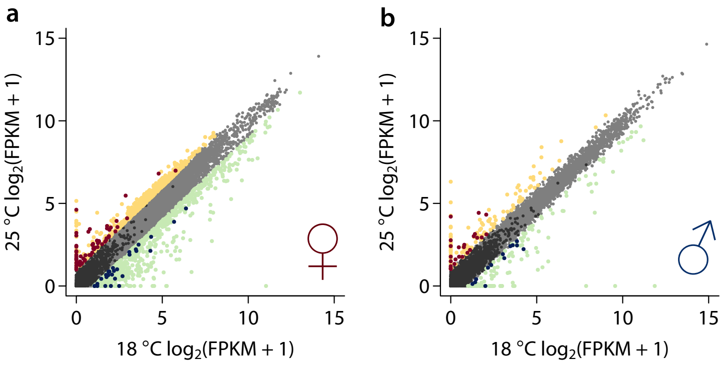

c

	♀	♂
Total (FPKM > 0 in either temperature)	19,787	20,905
Annotated	15,397	15,828
NTR	4,390	5,077
< 2 fold change	18,246	20,628
● Annotated	13,946	15,588
● NTR	4,300	5,040
18 °C/25 °C ≥ 2 fold	746	143
● Annotated	717	130
● NTR	29	13
25 °C/18 °C ≥ 2 fold	795	134
● Annotated	734	110
● NTR	61	24

Supplementary Fig. 1 | RNA-Seq annotation of the pooled RNA from DGRP lines
(**a**) Comparison of RNA-Seq expression estimates at 18 °C and 25 °C in females, colors of points indicate different classes of genes as indicated in (**c**). (**b**) Comparison of RNA-Seq expression estimates at 18 °C and 25 °C in males, colors of points indicate different classes of genes as indicated in (**c**). (**c**) Numbers of the different classes of genes based on expression comparison at 18 °C and 25 °C in females and males.

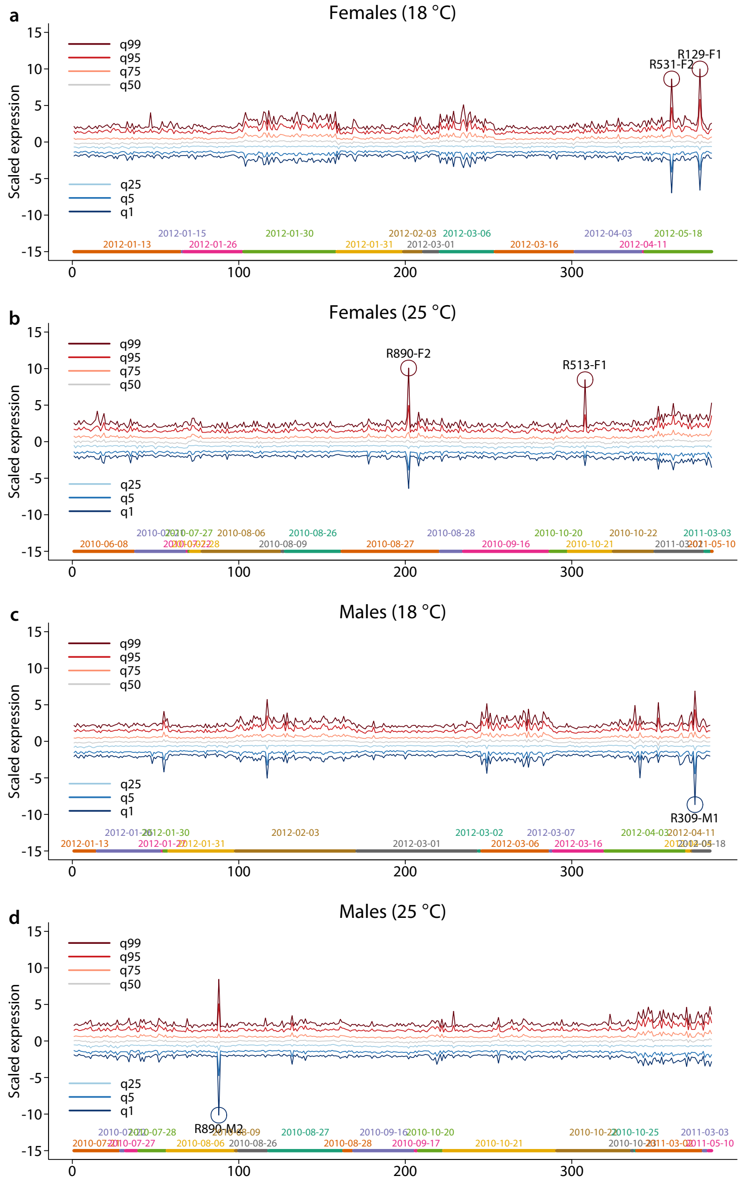

Supplementary Fig. 2 | Distribution of scaled gene expression across samples Within each sex and temperature, expression of genes was scaled across samples and the quantiles (1% [q1], 5% [q5], 25% [q25], 50% [q50], 75% [q75], 95% [q95], 99% [q99]) are visualized within each batch. Samples are arranged according to their array scan date, outliers are circled and annotated. (**a**) Females at 18 °C, (**b**) Females at 25 °C, (**c**) Males at 18 °C, (**d**) Males at 25 °C.

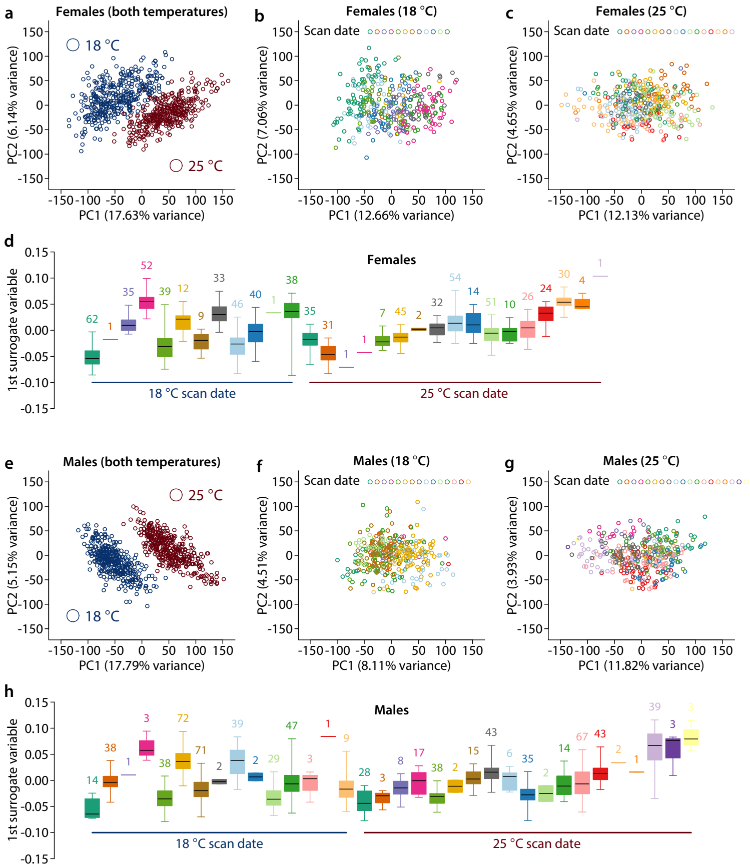

Supplementary Fig. 3 | Principal component analysis (PCA) and surrogate variable analysis (SVA) of gene expression (**a**) Principal component analysis in females combining samples from both 18 and 25 °C. (**b**) Principal component analysis in females at 18 °C. (**c**) Principal component analysis in females at 25 °C. (**d**) Variation in the first surrogate variable within and between the different array scan dates in females. (**e** - **h**) Same analyses but in males. Sample sizes are indicated above box plots in (**d**) and (**h**). For each box plot, the whiskers represent maximum and minimum values, box represents inter-quartile range and the middle line median.

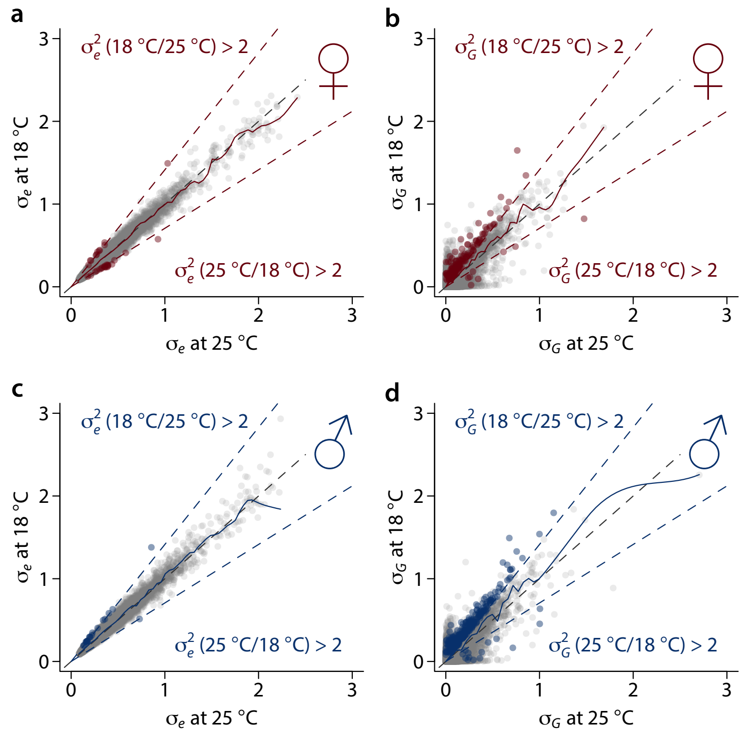

Supplementary Fig. 4 | Decanalization and canalization of genetic variance of gene expression at 18 °C without adjusting for *Wolbachia*. Same analyses as in **Fig. 2** except that *Wolbachia* was not adjusted.

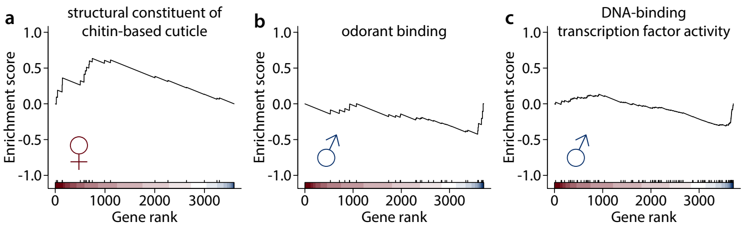

Supplementary Fig. 5 | Significant GO terms for the GSEA of decanalization/canalization. Trace plots for the three Gene Ontology (GO) terms (**a**) structural constituent of chitin-based cuticle enriched for genes of higher decanalization in females, (**b**) odorant binding enriched for genes of lower decanalization in males, and (**c**) DNA-binding transcription factor activity enriched for genes of lower decanalization in males.
