# Supplementary material for: Genotype by environment interaction for gene expression in Drosophila melanogaster
Source: Nat Commun. 2020 Oct 28;11:5451. doi: 10.1038/s41467-020-19131-y (PMC7595129; doi:10.1038/s41467-020-19131-y)
Supplement: Supplementary file 3 — Reporting Summary [file 41467_2020_19131_MOESM3_ESM.pdf]

nature research

Corresponding author(s): Wen Huang, Trudy Mackay

Last updated by author(s): Sep 12, 2020

Reporting Summary

Nature Research wishes to improve the reproducibility of the work that we publish. This form provides structure for consistency and transparency in reporting. For further information on Nature Research policies, see our Editorial Policies and the Editorial Policy Checklist.

Statistics

For all statistical analyses, confirm that the following items are present in the figure legend, table legend, main text, or Methods section.

n/a	Confirmed	
☐	☒	The exact sample size (n) for each experimental group/condition, given as a discrete number and unit of measurement
☐	☒	A statement on whether measurements were taken from distinct samples or whether the same sample was measured repeatedly
☐	☒	The statistical test(s) used AND whether they are one- or two-sided<br>*Only common tests should be described solely by name; describe more complex techniques in the Methods section.*
☐	☒	A description of all covariates tested
☐	☒	A description of any assumptions or corrections, such as tests of normality and adjustment for multiple comparisons
☐	☒	A full description of the statistical parameters including central tendency (e.g. means) or other basic estimates (e.g. regression coefficient) AND variation (e.g. standard deviation) or associated estimates of uncertainty (e.g. confidence intervals)
☐	☒	For null hypothesis testing, the test statistic (e.g. F, t, r) with confidence intervals, effect sizes, degrees of freedom and P value noted<br>*Give P values as exact values whenever suitable.*
☒	☐	For Bayesian analysis, information on the choice of priors and Markov chain Monte Carlo settings
☒	☐	For hierarchical and complex designs, identification of the appropriate level for tests and full reporting of outcomes
☐	☒	Estimates of effect sizes (e.g. Cohen's d, Pearson's r), indicating how they were calculated

Our web collection on statistics for biologists contains articles on many of the points above.

Software and code

Policy information about availability of computer code

Data collection	No software was used for the data collection.
Data analysis	The analyses were described in the methods section of the manuscript. All analysis codes are available in the GitHub repository (https://github.com/qgg-lab/dgrp-plasticity-eqtl). These codes are mostly scripts (R, bash, perl) using existing softwares. The commands provided as well as description in the Methods include version information of the softwares.

For manuscripts utilizing custom algorithms or software that are central to the research but not yet described in published literature, software must be made available to editors and reviewers. We strongly encourage code deposition in a community repository (e.g. GitHub). See the Nature Research guidelines for submitting code & software for further information.

Data

Policy information about availability of data

All manuscripts must include a data availability statement. This statement should provide the following information, where applicable:

- Accession codes, unique identifiers, or web links for publicly available datasets
- A list of figures that have associated raw data
- A description of any restrictions on data availability

- This study generated two new data sets. The RNA-Seq dataset was deposited to SRA (PRJNA615927) while the microarray data were deposited to ArrayExpress (E-MTAB-8953). These data have no restrictions and will be released immediately upon publication.
- Additional datasets used by this study were available publicly, including a RNA-Seq dataset (GEO: GSE67505) and microarray dataset (ArrayExpress: E-MTAB-3216).
- All data required to reproduce the figures and tables are available at the GitHub repository (https://github.com/qgg-lab/dgrp-plasticity-eqtl).

Field-specific reporting

Please select the one below that is the best fit for your research. If you are not sure, read the appropriate sections before making your selection.

☐ Life sciences ☐ Behavioural & social sciences ☒ Ecological, evolutionary & environmental sciences

For a reference copy of the document with all sections, see nature.com/documents/nr-reporting-summary-flat.pdf

Ecological, evolutionary & environmental sciences study design

All studies must disclose on these points even when the disclosure is negative.

Study description	This study exposed a population of inbred Drosophila melanogaster strains to two different temperatures. Two biological replicates for each sex and temperature. The design is a completely randomized factorial design within each temperature.
Research sample	This study uses laboratory samples suitable to address the questions. Samples are adult flies (females and males separately) from each of the DGRP strains. Whole body gene expression was measured.
Sampling strategy	Unbiased and randomized sampling. There was no selection on sampling, the entire DGRP was sampled.
Data collection	For the low temperature treatment, all DGRP strains were reared on cornmeal-molasses-agar medium at 18 °C with 60-75% relative humidity and a 12-h light/dark cycle. For each DGRP line, we collected 25 mated female or 40 mated male 3-5 day old flies to constitute one biological replicate for each sex. Collection was performed between 1 and 3PM consistently to account for circadian rhythm in gene expression and the flies were immediately frozen in liquid nitrogen before they were sorted. We collected two biological replicates per sex for each of 185 DGRP lines.
Timing and spatial scale	Sample collection was performed in the laboratory. To eliminate influence of circadian rhythm on gene expression, all collection was performed between 1 and 3PM consistently.
Data exclusions	To eliminate outliers, several samples were excluded based on their expression with pre-determined criteria. Samples with 99% quantile of scaled expression greater than 5 were excluded.
Reproducibility	Not applicable. The replication was used to estimate variance components, not for reproducing certain results per se.
Randomization	Sampling and microarray library preparation were completely randomized. The order of sampling and library preparation was randomized. No additional allocation to experimental groups was performed.
Blinding	The sample collection procedure must have IDs of the strains thus blinding was not possible. Randomization effectively results in blinding in subsequent steps.

Did the study involve field work? ☐ Yes ☒ No

Reporting for specific materials, systems and methods

We require information from authors about some types of materials, experimental systems and methods used in many studies. Here, indicate whether each material, system or method listed is relevant to your study. If you are not sure if a list item applies to your research, read the appropriate section before selecting a response.

Materials & experimental systems

n/a	Involved in the study	
☒	☐	Antibodies
☒	☐	Eukaryotic cell lines
☒	☐	Palaeontology and archaeology
☐	☒	Animals and other organisms
☒	☐	Human research participants
☒	☐	Clinical data
☒	☐	Dual use research of concern

Methods

n/a	Involved in the study	
☒	☐	ChIP-seq
☒	☐	Flow cytometry
☒	☐	MRI-based neuroimaging

Animals and other organisms

Policy information about studies involving animals; ARRIVE guidelines recommended for reporting animal research

Laboratory animals	Drosophila melanogaster, strains from the DGRP, mated females and males 3-5 days old.
Wild animals	No wild animals were used.

Field-collected samples	No field collected samples were used.
Ethics oversight	Invertebrate animals, no ethics approval was required.

Note that full information on the approval of the study protocol must also be provided in the manuscript.
