# Supplementary material for: Genotype by environment interaction for gene expression in Drosophila melanogaster
Source: Nat Commun. 2020 Oct 28;11:5451. doi: 10.1038/s41467-020-19131-y (PMC7595129; doi:10.1038/s41467-020-19131-y)
Supplement: Supplementary file 4 — Description of Additional Supplementary Files [file 41467_2020_19131_MOESM4_ESM.pdf]

Description of Additional Supplementary Files

Supplementary Data 1
Summary of RNA-Seq and alignments.

Supplementary Data 2
Variance components and significance tests (P value adjusted using BenjaminiHochberg approach).

Supplementary Data 3
Gene set enrichment analysis (GSEA) of genetic decanalization and canalization (P value adjusted using Benjamini-Hochberg approach).

Supplementary Data 4
Gene set enrichment analysis (GSEA) of genotype by environment interaction (P value adjusted using Benjamini-Hochberg approach).

Supplementary Data 5
eQTL mapping and model selection results.

Supplementary Data 6
GO enrichment in module 1 of the female co-expression network (P value adjusted using Benjamini-Hochberg approach).

Supplementary Data 7
Network preservation statistics using the NetRep package in R. Supplementary Data 8. Comparison of GxE genes in this study (males) with genes in other studies.
