# Supplementary material for: Genotype by environment interaction for gene expression in Drosophila melanogaster
Source: Nat Commun. 2020 Oct 28;11:5451. doi: 10.1038/s41467-020-19131-y (PMC7595129; doi:10.1038/s41467-020-19131-y)
Supplement: Supplementary file 2 — Peer Review [file 41467_2020_19131_MOESM2_ESM.pdf]

Reviewer #1 (Remarks to the Author):

Huang et al. study heritable variation in gene expression between environments in the DGRP panel of inbred fruit fly lines. They determine the transcribed portion of the genome at two temperatures--18 and 25 degrees Celsius--by sequencing pooled RNA from a large number of fly genotypes. They then use tiling microarrays to obtain transcriptome-wide gene expression measurements for each genotype at each temperature. 25 degrees Celsius is the normal temperature used to grow this organism in the lab, enabling analysis of how heritable gene expression differs between the species' normal temperature and an atypical one. With these data, as well prior data for mutation accumulation (MA) lines, the authors explore relationships between heritable gene expression, the environment, and natural selection.

The authors find evidence for genotype by environment interaction (GEI) for a large number of genes, with thousands showing significant broad-sense heritability in both environments. A large portion of these same genes have an eQTL detected in both temperatures, allowing the authors to detect transcription factor binding sites (TFBSs) enriched among these eQTLS. The authors also identify modules of co-expressed genes with GEI and find correlations between stabilizing selection and network connectivity.

This was an interesting paper. The figures are aesthetically pleasing and the manuscript is well written. The work is high quality. I think there might be a good amount of novelty in this paper for the Drosophila research community and some of the insights about stabilizing selection and network connectivity could be of broad interest. This was not just another standard genetics of gene expression paper; the authors meaningfully attempt to answer more general questions about how evolution shapes the way organisms' genetics interact with their environments. If I had to criticize anything about this paper, it would just be that the most interesting and novel parts of the paper are a bit superficial. For example, regarding mechanism, although a connection to particular transcription factors is noted, generally there is not much discussion of what the coexpression modules represent functionally, why the system responds the way it does to temperature, and what types of genes and genetic variants are under selection. Presumably the mapping study performed here is high enough resolution to permit such inquiry.

Specific points:

-The terms canalization and decanalization are heavily used in this paper, but usage of these terms implies that the system has evolved to have low variability at 25 degrees Celsius and this canalization then gets perturbed at 18 degrees Celsius. How can this be assumed? Other terms that might depend less on invoking selection are preferable.

-MA lines were only generated at one temperature (this was done in past, published work). If the MA lines had been generated at 18 degrees Celsius, or at both temperatures, would any different results have been found?

-Very little is described about the molecular nature of identified QTLs, except for the enrichment for particular TFBSs.

-In Fig 6, there are many points with very high or low values [>=1E4 and <=1E-4]. Should these be included in this analysis? Why are this this way? There seems to be an excess of these points at low values. Are they skewing the correlation at all?

-Also, tied to Fig 6, I could not tell if only genes with GEI are shown or if all genes are shown. If all genes are shown, it might be good to demonstrate, likely in the supplement, that the pattern holds for GEI genes but not those genes without GEI. Or, just a sentence in the figure legend.

Reviewer #2 (Remarks to the Author):

The authors in 'Plasticity of Regulatory Variation in Gene Expression in Drosophila melanogaster' investigate the genetic architecture of regulatory variation by examining the gene expression of DGRP lines in two temperature regimes. They found some genes with GxE, mostly at 18°C. most of the eQTLs were also shared between the two temperatures, with enrichment for two transcription factors. They also did not find eQTLs to cause any disruption in gene expression network connectivity. This study addresses an important question in evolutionary biology: what is the role of plasticity in the genetic architecture of complex traits. The prevalence of phenotypic plasticity is interesting, although plasticity and even adaptation of plasticity has been investigated/shown before. At the current state of the manuscript, the results are presented without much attempt in explaining the effect of these findings in the fate of populations if they face a novel environment and have to adapt. After all, plasticity should facilitate adaptation of organisms to novel environments. I think the manuscript will benefit from interpretation of the findings in an evolutionary context.
Major comments:
1. This study will benefit from incorporating the invaluable genomic resources of DGRP. The authors have mapped eQTLs on around 2M SNPs. It would be interesting to see the characteristics of SNPs that cause such GxE, for example allele frequencies. Although the allelic frequencies in DGRP lines differ from natural populations but information for natural populations are available that can be used for comparison. In particular, since authors have tried to assess plasticity in 'low temperature' (note that 18°C is not quite low and some natural populations are exposed to much lower temperatures) it would be interesting to see whether such SNPs are adaptive and can be found in clines of D. melanogaster in North America and Australia (Machado et al., 2016; Reinhardt et al. 2014). Also, the authors can check whether the genes with plastic expression or eQTLs are also differentially expressed in natural populations as well (Hutter et al. 2008).
Machado HE, et al. (2016) Comparative population genomics of latitudinal variation in Drosophila simulans and Drosophila melanogaster. Mol. Ecol. 25, 723–740.
Reinhardt JA, Kolaczkowski B, Jones CD, Begun DJ, Kern AD. (2014) Parallel geographic variation in Drosophila melanogaster. Genetics 197, 361–73.
Hutter S, Saminadin-peter SS, Stephan W, Parsch J. (2008) Gene expression variation in African and European populations of Drosophila melanogaster. Genome Biol. 2008; 9(1):1–15.
Comparison of the findings of this study to natural populations would determine whether the plastic response observed here could potentially be used for adaptation of populations. In GWAS many loci contribute to a complex trait but most alleles are deleterious or due to constraints (e.g. pleiotropy) are unable to respond to selection. The observed plasticity in this study might also be deleterious, unless fitness data is available. The authors can discuss the implications of their results in potential adaptive scenarios.
2. Authors here investigate the genetic architecture of regulatory variation in gene expression (it will make the manuscript easier to read if, when applicable, term 'plasticity' is used, see comment 3) by studying the gene expression in DGRP inbred lines in only two temperature regimes. Another study cited here (Chen et al. 2015) have found much larger fraction of genes (around 80%) to be differentially expressed across 4 temperature regimes. Inclusion of more temperature regimes might have result in identification of 1) more/different genes with genetic variation in expression and 2) some negative sign changes might also be observed. Thus, it should be discussed in the discussion that results and conclusions are restricted by availability of gene expression from 2 environments only.

3. I decided to include this comment in major comments because it really affects the readability of the manuscript. To improve the readability, I urge the authors to rephrase some parts:
a) Line 67-68/Line 180: Does 'to understand the plasticity of the genetic architecture of gene expression' mean that the aim of study is to understand the plasticity of gene expression. If yes, then 'genetic architecture' can be deleted, if the authors mean otherwise, it should be clarified. In line 194, 'plasticity of regulatory variation' makes total sense without the use of genetic architecture. This style

can be adapted throughout the manuscript.
b) Line130: It seems that definition of genetic variation here is somehow different from the one used in population genetics. Is 'genetic variation' in gene expression the same as variation in gene expression? It seems that genetic variation is used for referring to 'between-line variation in gene expression'. Line 297-298: does it mean that the expression of many genes changed between the two temperatures? (in this case 'genetic variation' can be substituted with 'expression') Or did between-line gene expression variation changed?
c) The same is for the use of 'genetic variation' in line 148. It will help the readers to have a definition of 'genetic variation' the same way canalization and decanalization are defined.
d) Line 149-150: Is 'genetic basis of genotype by environment interaction in gene expression' the 'the genetic basis of gene expression plasticity'? if not, how does it differ?
e) Lines 168-171: These two sentences have the same meaning; you can use one only. When you mention 'genetic architecture' it implies heritability.

Minor comments:
1. The authors find more decanalization at 18°C compared to 25°C. This is in contrast to another study cited here (Chen et al. 2015) where 2 different lines had the most similar gene expression at 18°C. It worth discussing whether the observed response is due to 18C being a 'low' temperature for development of flies, or the variation in gene expression is amplified in the novel temperature compared to the temperature that lines are kept at, whether 18°C or 25°C.

2. RNA-seq data has been used to only reconstruct transcript models, why has it not been used for estimation of gene expression?

3. Line 206-207: do authors mean that the majority of eQTLs were shared between the two environments? please rephrase the sentence to clarify the take-home-message.

4. For majority of genes, the variation in gene expression were in the same direction at 18°C and 25°C temperature regimes (Lines 223). Given the direction of gene expression variation, the ability to predict the expression of genes in one environment using the expression in the other is quite expected (line 224-229). Isn't this trivial? If it was intended to make another conclusion, it should be clarified.

5. Line 240: Enrichment of eQTLs for two transcription factors is emphasized in the abstract, it would be good to elaborate whether the function of chinmo and eve are related to the novel temperature DGRP lines experienced.

6. Are the eQTLs enriched for specific tissues?

7. Line 317-318, the model the authors referring to seem to be omnigenic model (Boyle et al. 2017).
Boyle EA, Li YI, Pritchard JK (2017). An expanded view of complex traits: from polygenic to omnigenic. Cell. 169(7): 1177–1186.

Reviewer #3 (Remarks to the Author):

The study "Plasticity of Regulatory Variation in Gene Expression in Drosophila melanogaster" explores one of the most complex concepts in evolutionary biology – gene by environment interactions. The authors set out to map and describe the genetic variation underlying the variation of plasticity. This study is a feat of data production with an impressive set of genome wide transcriptomic data for 185 lines, male and females, all in duplicate. More than simply producing it, the authors use the data aptly to explore some truly complex questions, such as genetic variation of genome-wide GxE interactions. The results are interesting and will be of relevance to the broad readership, but more so to the fields

of quantitative genetics, evolutionary biology and ecology.
There are several aspects of this study that will be of interest to the field, mainly the impact of genetic variation on plastic responses to new environment, an important phenomenon we need to understand in order to someday be able to predict phenotype from genotypes outside the controlled environment of the laboratory. Although some of these findings have been previously reported (e.g. variable gene expression depending on genotype and environment interaction), this study stands out for various reasons, mainly the sheer number of genotypes tested, and the subsequent depth of analyses, both of which allowed for, to my knowledge, most comprehensive characterization of genetic architecture of genome-wide GXE interactions for transcriptomic data (at least in Drosophila). This also makes the conclusions drawn here more generalizable than in previous work done on a limited set of genotypes.
The authors also use the data to explore which eQTL that may underlie regulation of GxE and how, which is an exciting prospect for studying mechanisms behind GxE interactions. They also test co-expression network robustness and report that such networks are maintained upon environmental change - this study is a composite of more than a few interesting results.
On a personal note, I have been wondering why such a study has not been done yet, and I have even already contemplated several times of rolling up sleeves to do it. I am glad it has now been done!

I do have some minor comments that need to be addressed, mostly regarding terminology:

1. The concept of canalization has historically been really poorly defined and as such causes many disputes among research groups. I suggest you make sure to define canalization as well as possible, especially with respect to using polymorphic lines for this study. For example, there has been some excellent work coming from Mark Siegal's lab showing that most of the genetic mutational buffering i.e. canalization is nothing but genetic background effect (eg https://doi.org/10.1371/journal.pbio.0060264 or https://doi.org/10.1371/journal.pgen.1003733), but this all depends on how you define robustness to mutation, and where the mutations are. If genetic canalization is being invoked it may be good to at least discuss the effects of variation at a single locus on different genetic backgrounds vs genetic variation at a locus while the rest of the genetic background is the same across lines. Similarly, when referring to canalization, please note if you mean genetic or environmental decanalization throughout the manuscript to avoid confusion.
2. I think that often when referring to genetic variance you write "genetic variation" which may be confusing because when one hears "genetic variation" DNA variation across lines is the first thing that comes to mind and not the variation in gene expression across lines. This may be confusing.
L55: I think that because G x E is such a common abbreviation in the field using it over GEI may make the study more readable.
L21,57(and throughout the manuscript) : The term "plastic genetic architecture" does not sound right to me. Usually in the field plasticity refers to the phenotype and not to genetic architecture; it is not the genotype that is changing but more that the expression of the genotype is variable across environments. I see why in the case of GxE you could maybe call it plasticity of the genetic architecture, but this kind of nomenclature adds another layer of complexity on an already complex concept. It's kind of like genetic plasticity of plasticity; genetic variation or genetic variability would be better suited.
L19 (and throughout the manuscript): Because GxE refers to not just temperature but different rearing batches please change to "when flies developed at different environments". I think it was a fair point that you have highlighted this in L110, but I would also suggest to still be more careful at wording when declaring causative factors for change in variance. For example, in cases like when you find genetic decanalization at 18C and canalization at 25C, which may seem to starkly contradict the results by Chen et al. 2015, this difference may simply be a consequence of variance increasing due to other, unknown factor in this batch.
L310 How do you reconcile this result with recent report of highly polygenic architecture in adaptation to novel thermal environments by Barghi et al. 2019?
L148 For a reader used to the concept of canalization and plasticity this is a good walk-through interpreting or disentangling one from another. However, I believe a visual representation of these processes may substantially help the naïve reader and help you make your point! Maybe consider

adding a schematic figure depicting the different concepts, perhaps even in the main text. For me, it was also sometimes difficult to follow which variance is being considered and in which context, so in case you opt out of a schematic figure, it would benefit the manuscript to try to reword this paragraph with a naïve reader in mind.
L163 change to “genotype by environment interaction”

Fig 2
In the caption point out that the significant points are blue, as well as red points.
Is there a particular reason why LOESS is used?

Fig4
a) This is a very confusing figure, needs more explanation in caption at least, for example I was totally lost what the different numbers are and how they refer to the text where Fig 3 was referenced.
g) why permute over the whole set instead of within 25 C only?

Fig 6
I have to say that the correlation plots although significant, seem unconvincingly flat. Is the slight positive slope driven by the cluster of points with very low stabilizing selection values?

## Response to reviewers

Reviewer #1 (Remarks to the Author):

Huang et al. study heritable variation in gene expression between environments in the DGRP panel of inbred fruit fly lines. They determine the transcribed portion of the genome at two temperatures--18 and 25 degrees Celsius--by sequencing pooled RNA from a large number of fly genotypes. They then use tiling microarrays to obtain transcriptome-wide gene expression measurements for each genotype at each temperature. 25 degrees Celsius is the normal temperature used to grow this organism in the lab, enabling analysis of how heritable gene expression differs between the species' normal temperature and an atypical one. With these data, as well prior data for mutation accumulation (MA) lines, the authors explore relationships between heritable gene expression, the environment, and natural selection.

The authors find evidence for genotype by environment interaction (GEI) for a large number of genes, with thousands showing significant broad-sense heritability in both environments. A large portion of these same genes have an eQTL detected in both temperatures, allowing the authors to detect transcription factor binding sites (TFBSs) enriched among these eQTLS. The authors also identify modules of co-expressed genes with GEI and find correlations between stabilizing selection and network connectivity.

This was an interesting paper. The figures are aesthetically pleasing and the manuscript is well written. The work is high quality. I think there might be a good amount of novelty in this paper for the Drosophila research community and some of the insights about stabilizing selection and network connectivity could be of broad interest. This was not just another standard genetics of gene expression paper; the authors meaningfully attempt to answer more general questions about how evolution shapes the way organisms' genetics interact with their environments. If I had to criticize anything about this paper, it would just be that the most interesting and novel parts of the paper are a bit superficial. For example, regarding mechanism, although a connection to particular transcription factors is noted, generally there is not much discussion of what the coexpression modules represent functionally, why the system responds the way it does to temperature, and what types of genes and genetic variants are under selection. Presumably the mapping study performed here is high enough resolution to permit such inquiry.

We thank the reviewer for the enthusiasm and the constructive feedback.

Specific points:

-The terms canalization and decanalization are heavily used in this paper, but usage of these terms implies that the system has evolved to have low variability at 25 degrees Celsius and this canalization then gets perturbed at 18 degrees Celsius. How can this be assumed? Other terms that might depend less on invoking selection are preferable.

This is an important point and we thank the reviewer for the opportunity to clarify. As we noted in the paper, the terms canalization and decanalization are relative and depend on the chosen baseline or

reference point. We arbitrarily chose 25 °C as the reference point for the ease of discussion and this by no means implies that it has lower variability. We have now clarified what the reference points are whenever these terms are used (see for example L22-23, L138-139).

-MA lines were only generated at one temperature (this was done in past, published work). If the MA lines had been generated at 18 degrees Celsius, or at both temperatures, would any different results have been found?

We thank the reviewer for this insightful and thought-provoking question. Although we cannot prove this experimentally, we believe the results will still hold for the following reasons. In particular, we think the correlation between gene connectivity and strength of stabilizing selection (Vm/Vg) is robust regardless of the environments in which the mutation accumulation (MA) lines are generated.

1) The mutation accumulation lines experienced severe population bottlenecks, and hence only strongly fitness altering mutations (e.g. lethal) are selected for or against. In addition, the spontaneous mutations were randomly distributed across the genome. Therefore, although the mutation accumulation process happened at 25 degrees only, the mutational profiles would be similar had the lines experienced novel or fluctuating environments.
2) The standing variation in the DGRP, on the other hand, is a reflection of what the local North Carolina fly population had experienced and what had the abilities to shape the genetic variation, including environmental factors such as fluctuating temperatures.
3) The potential problem lies in the environment in which the Vm and Vg are measured, especially if they change in different directions or magnitudes when the environment changes. Importantly, all three quantities involved in estimating the correlation, including Vm, Vg, and gene expression, were obtained at 25 °C. The correlation between gene connectivity and strength of stabilizing selection estimated at 25 °C thus should be considered a representative environment and strictly speaking should be specific to this environment.
4) However, because Vg is shaped by the historical environments of the DGRP in the wild and given the wide range of temperature in North Carolina, the correlation would diminish if it was environment specific. In other words, we may actually have underestimated the correlation by estimating Vm/Vg using Vg that was shaped by heterogeneous environments. Nevertheless, despite the relatively small magnitude of correlation (which could be partly explained by the heterogeneity of historical environments), the signal was highly significant. Therefore we believe the correlation is robust to the heterogeneity of environment.

-Very little is described about the molecular nature of identified QTLs, except for the enrichment for particular TFBSs.

Given the genomic nature of the paper, we mostly make only global characterization of patterns in the data. The global patterns can answer important fundamental questions, and we intentionally avoided discussing specific genes, variants, or mechanisms, which require much more supporting data. We agree with the reviewer that this makes the paper seem superficial, but they are starting points of many exciting follow-up studies.

Regarding the molecular nature of the identified QTLs, many have been previously characterized in

eQTL mapping work at 25 °C in Huang et al. 2005 and Everett et al. 2020 (references in the manuscript), including enrichment of eQTLs around gene boundaries and in different genomic regions. These are common features about eQTLs in any environment. The same can be said for general features including functional enrichment among co-expression modules, as the reviewer pointed out above.

In this paper, we specifically focus on the differences between the two temperatures. The TFBSs enrichment is an obvious analysis, for which we can perform robust permutation-based approach to attach statistical significance. Regarding the two transcription factors mentioned, we added one possible mechanism by which they may regulate environment specific regulatory variation in light of a recent report that antennal sensory neurons underly absolute temperature sensing in Drosophila (L337-341).

-In Fig 6, there are many points with very high or low values [>=1E4 and <=1E-4]. Should these be included in this analysis? Why are this this way? There seems to be an excess of these points at low values. Are they skewing the correlation at all?

We capped those values because they were too small or too large to fit the plot. These were mostly the effects of the variance components being close to zero for either Vg or Vm. These points won't skew the correlation because we used rank correlation (Spearman) for exactly this reason. In addition, although there appears to be many such points, they only account for about 3% of all points displayed.

-Also, tied to Fig 6, I could not tell if only genes with GEI are shown or if all genes are shown. If all genes are shown, it might be good to demonstrate, likely in the supplement, that the pattern holds for GEI genes but not those genes without GEI. Or, just a sentence in the figure legend.

We thank the reviewer for this suggestion. We now demonstrate genes with or without significant GEI (termed GxE now following suggestion by another reviewer) by color coding them. In the caption, we indicate what the correlations and P values are when limiting to each group of genes (Figure 6), all of which remain significant.

Reviewer #2 (Remarks to the Author):

The authors in 'Plasticity of Regulatory Variation in Gene Expression in Drosophila melanogaster' investigate the genetic architecture of regulatory variation by examining the gene expression of DGRP lines in two temperature regimes. They found some genes with GxE, mostly at 18°C. most of the eQTLs were also shared between the two temperatures, with enrichment for two transcription factors. They also did not find eQTLs to cause any disruption in gene expression network connectivity. This study addresses an important question in evolutionary biology: what is the role of plasticity in the genetic architecture of complex traits. The prevalence of phenotypic plasticity is interesting, although plasticity and even adaptation of plasticity has been investigated/shown before. At the current state of the manuscript, the results are presented without much attempt in explaining the effect of these findings in the fate of populations if they face a novel environment and have to adapt.

After all, plasticity should facilitate adaptation of organisms to novel environments. I think the manuscript will benefit from interpretation of the findings in an evolutionary context.

We thank the reviewer for appreciating the importance of this work and the constructive feedback.

Major comments:

1. This study will benefit from incorporating the invaluable genomic resources of DGRP. The authors have mapped eQTLs on around 2M SNPs. It would be interesting to see the characteristics of SNPs that cause such GxE, for example allele frequencies. Although the allelic frequencies in DGRP lines differ from natural populations but information for natural populations are available that can be used for comparison. In particular, since authors have tried to assess plasticity in 'low temperature' (note that 18°C is not quite low and some natural populations are exposed to much lower temperatures) it would be interesting to see whether such SNPs are adaptive and can be found in clines of D. melanogaster in North America and Australia (Machado et al., 2016; Reinhardt et al. 2014). Also, the authors can check whether the genes with plastic expression or eQTLs are also differentially expressed in natural populations as well (Hutter et al. 2008).

Machado HE, et al. (2016) Comparative population genomics of latitudinal variation in Drosophila simulans and Drosophila melanogaster. Mol. Ecol. 25, 723–740.

Reinhardt JA, Kolaczkowski B, Jones CD, Begun DJ, Kern AD. (2014) Parallel geographic variation in Drosophila melanogaster. Genetics 197, 361–73.

Hutter S, Saminadin-peter SS, Stephan W, Parsch J. (2008) Gene expression variation in African and European populations of Drosophila melanogaster. Genome Biol. 2008; 9(1):1–15.

Comparison of the findings of this study to natural populations would determine whether the plastic response observed here could potentially be used for adaptation of populations. In GWAS many loci contribute to a complex trait but most alleles are deleterious or due to constraints (e.g. pleiotropy) are unable to respond to selection. The observed plasticity in this study might also be deleterious, unless fitness data is available. The authors can discuss the implications of their results in potential adaptive scenarios.

We thank the reviewer for pointing out relevant literature that we did not discuss. We have now added a discussion on the overlap between genes identified in this study and genes identified in other studies that showed evidence of adaptive evolution or GxE due to temperature. We completely agree with the reviewer that the evolutionary implications of thermal plasticity can be very interesting to consider!

Unfortunately, the Machado et al. 2016 and Reinhardt et al. 2014 papers were global characterizations of patterns of seasonally and spatially varying SNPs but did not contain identities of SNPs that showed temporal and spatial variation. We also did not find other studies that published such SNPs along with the papers to allow for comparison.

Nevertheless, we were able to perform an analysis of the intersection between genes that were significant in our study for GxE and these previous studies (all male expression):

1) significant in the Hutter et al. 2008 study for differential expression between African (tropical) and European (temperate) populations.
2) significant for GxE in the Levine et al. 2011 study
3) significant at either of two temperatures (21 °C and 29 °C) for differential expression between a Panama (tropical) and a Maine (temperate) population (Zhao et al. 2015)

These have been added to the discussion of the paper (L319-327). However, we could only make an ad-hoc assessment of overlap without statistical significance for enrichment because none of the studies contained information for the full sets of genes to allow for such inference.

Although these analyses weren't very fruitful, we would like to emphasize that our study focuses more on gene expression as a model and less on the phenotypic response from low temperature, which as the reviewer pointed out, isn't really low. Connecting the findings with data from natural populations is very appealing, and we believe after publication and release of the full dataset, we and others will be able to utilize this resource when the right data come along.

We very much appreciate this comment from the reviewer, which would have made this paper more impactful if we were able to connect the findings with adaptation in the wild. Unfortunately, this remains a very difficult problem and the right experiments are yet to be done. We point this limitation out in our discussion.

2. Authors here investigate the genetic architecture of regulatory variation in gene expression (it will make the manuscript easier to read if, when applicable, term 'plasticity' is used, see comment 3) by studying the gene expression in DGRP inbred lines in only two temperature regimes. Another study cited here (Chen et al. 2015) have found much larger fraction of genes (around 80%) to be differentially expressed across 4 temperature regimes. Inclusion of more temperature regimes might have result in identification of 1) more/different genes with genetic variation in expression and 2) some negative sign changes might also be observed. Thus, it should be discussed in the discussion that results and conclusions are restricted by availability of gene expression from 2 environments only.

Unlike the Chen et al. study, which found genes responding to different temperatures for a single genotype (F1 hybrid between Oregon R and Samarkand), we did not attempt to identify such genes because of confounding by batch effect. Instead, we identified genes that responded differently across the genetically diverse lines (GxE). Thus the numbers are not directly comparable. Levine et al. 2011 (cited in the paper) found genes that exhibited GxE in response temperature change with a different design, but they used a threshold of top 300 genes rather than basing the inference on a statistical test.

That being said, we agree with the reviewer that we may have underestimated the extent of GxE because only two environments were considered. We have now pointed out the limitation of the study with respect to the small number of environments sampled in the manuscript (L315-318).

3. I decided to include this comment in major comments because it really affects the readability of the manuscript. To improve the readability, I urge the authors to rephrase some parts:

We thank the reviewer for the opportunity to clarify these points. Following this suggestion, we have now adopted a more consistent terminology with clear definitions.

a) Line 67-68/Line 180: Does 'to understand the plasticity of the genetic architecture of gene expression' mean that the aim of study is to understand the plasticity of gene expression. If yes, then 'genetic architecture' can be deleted, if the authors mean otherwise, it should be clarified. In line 194, 'plasticity of regulatory variation' makes total sense without the use of genetic architecture. This style can be adapted throughout the manuscript.

Following the reviewer's suggestion, we have gone through the manuscript to clarify in all places where ambiguity is possible. In particular, we have tried to adopt a more consistent style throughout the manuscript. We did make some judgement calls when the choice of a particular term flows better within the context.

In this paper, these four terms are equivalent (revised term in parentheses, does not change any analyses or conclusions):

plasticity (environmental response) of the genetic architecture of gene expression
genetic architecture of the plasticity of gene expression
plasticity (environmental response) of regulatory (genetic) variation
GxE of gene expression

The term "plasticity (environmental response) of regulatory variation" is especially tricky because "regulatory (genetic) variation" = "genetic architecture of gene expression" = "the part of gene expression variation that is genetically regulated". The plasticity (environmental response) of the genetic architecture of gene expression therefore is not the same as plasticity of gene expression; the latter is change in response to environmental change and may or may not vary across different genotypes within or between species.

We defined these terms and explained their equivalency (L154-170). We added illustrative diagrams to support these definitions (Figure 2 and 3). We made this unifying effort because all terms are widely used in the literature in different contexts. We believe our revision has made these sufficiently clear.

b) Line130: It seems that definition of genetic variation here is somehow different from the one used in population genetics. Is 'genetic variation' in gene expression the same as variation in gene expression? It seems that genetic variation is used for referring to 'between-line variation in gene expression'. Line 297-298: does it mean that the expression of many genes changed between the two temperatures? (in this case 'genetic variation' can be substituted with 'expression') Or did between-line gene expression variation changed?

We thank the reviewer for the opportunity to clarify. We have now changed 'genetic variation' to

‘genetic variance’ to specifically refer to between-line variance in gene expression. Line 297-298 (now L309-310) means between-line gene expression variance changed.

c) The same is for the use of ‘genetic variation’ in line 148. It will help the readers to have a definition of ‘genetic variation’ the same way canalization and decanalization are defined.

We have now changed ‘genetic variation’ to ‘genetic variance’ and hopefully this clarifies the definition.

d) Line 149-150: Is ‘genetic basis of genotype by environment interaction in gene expression’ the ‘the genetic basis of gene expression plasticity’? if not, how does it differ?

It is. We changed this to “environmental response of the genetic architecture of gene expression” to be consistent with terminologies adopted throughout the paper. We chose this term in this place because we mapped eQTLs in the two temperatures and then compared their identities and effects so this term is a precise characterization of the analyses performed.

e) Lines 168-171: These two sentences have the same meaning; you can use one only. When you mention ‘genetic architecture’ it implies heritability.

They indeed are. They were meant to emphasize the equivalency.

Minor comments:

1. The authors find more decanalization at 18°C compared to 25°C. This is in contrast to another study cited here (Chen et al. 2015) where 2 different lines had the most similar gene expression at 18°C. It worth discussing whether the observed response is due to 18C being a ‘low’ temperature for development of flies, or the variation in gene expression is amplified in the novel temperature compared to the temperature that lines are kept at, whether 18°C or 25°C.

The Chen et al. PLOS Genetics 2015 study identified differentially expressed genes between two lines at 13 °C, 18 °C, 23 °C, and 29 °C and observed that at 18 °C there was strong genetic canalization, *i.e.* very few allelic effects at this temperature relative to others. However, there is a key difference between the designs of the two studies. We defined genetic variance as the between-line variance across 185 lines while Chen et al. defined allelic effects as the difference between two lines. It is entirely possible that there exists a pair or pairs of lines in the DGRP in which there are more differentially expressed genes at 18 °C than at 25 °C. We think it is unlikely that the ‘low’ temperature had led to the low variation in gene expression because there were more differentially expressed genes at an even lower temperature at 13 °C in the Chen et al. 2015 study.

2. RNA-seq data has been used to only reconstruct transcript models, why has it not been used for estimation of gene expression?

The study was started at a time when RNA-Seq was still quite expensive (ca. 2012-2013). We circumvented this challenge by combining RNA-Seq for transcript model reconstruction and tiling

microarrays (can also estimate expression of unannotated genes), taking good advantage of both technologies.

3. Line 206-207: do authors mean that the majority of eQTLs were shared between the two environments? please rephrase the sentence to clarify the take-home-message.

Not quite. The sharing of eQTLs was limited to genes whose eQTLs can be mapped in both environments. There were still genes with eQTLs in only one environment. We have clarified these descriptions in the paper, especially with respect to Figure 4a (also see caption of Figure 4).

4. For majority of genes, the variation in gene expression were in the same direction at 18°C and 25°C temperature regimes (Lines 223). Given the direction of gene expression variation, the ability to predict the expression of genes in one environment using the expression in the other is quite expected (line 224-229). Isn't this trivial? If it was intended to make another conclusion, it should be clarified.

It is an expected result but the implication isn't trivial. There have been studies that use eQTLs mapped in reference populations to perform association tests between predicted gene expression and phenotypes (e.g. PrediXcan). This result suggests that it cannot always be assumed that mapped eQTLs can predict expression well, especially in the presence of GxE. We have made this additional point in the discussion (L331-334).

5. Line 240: Enrichment of eQTLs for two transcription factors is emphasized in the abstract, it would be good to elaborate whether the function of chinmo and eve are related to the novel temperature DGRP lines experienced.

We have now expanded discussion on a possible mechanism of how their binding sites may regulate the environmentally responsive regulatory variation (L337-341).

6. Are the eQTLs enriched for specific tissues?

We could not infer enrichment for eQTLs because the expression was whole-body expression.

7. Line 317-318, the model the authors referring to seem to be omnigenic model (Boyle et al. 2017).

Boyle EA, Li YI, Pritchard JK (2017). An expanded view of complex traits: from polygenic to omnigenic. Cell. 169(7): 1177–1186.

We thank the reviewer for pointing out this connection. The two models differ in the directions of information flow but have a connection that we now make in the paper (L352-357).

Reviewer #3 (Remarks to the Author):

The study "Plasticity of Regulatory Variation in Gene Expression in Drosophila melanogaster" explores one of the most complex concepts in evolutionary biology – gene by environment

interactions. The authors set out to map and describe the genetic variation underlying the variation of plasticity. This study is a feat of data production with an impressive set of genome wide transcriptomic data for 185 lines, male and females, all in duplicate. More than simply producing it, the authors use the data aptly to explore some truly complex questions, such as genetic variation of genome-wide GxE interactions. The results are interesting and will be of relevance to the broad readership, but more so to the fields of quantitative genetics, evolutionary biology and ecology.

There are several aspects of this study that will be of interest to the field, mainly the impact of genetic variation on plastic responses to new environment, an important phenomenon we need to understand in order to someday be able to predict phenotype from genotypes outside the controlled environment of the laboratory. Although some of these findings have been previously reported (e.g. variable gene expression depending on genotype and environment interaction), this study stands out for various reasons, mainly the sheer number of genotypes tested, and the subsequent depth of analyses, both of which allowed for, to my knowledge, most comprehensive characterization of genetic architecture of genome-wide GXE interactions for transcriptomic data (at least in Drosophila). This also makes the conclusions drawn here more generalizable than in previous work done on a limited set of genotypes. The authors also use the data to explore which eQTL that may underlie regulation of GxE and how, which is an exciting prospect for studying mechanisms behind GxE interactions. They also test co-expression network robustness and report that such networks are maintained upon environmental change - this study is a composite of more than a few interesting results.

On a personal note, I have been wondering why such a study has not been done yet, and I have even already contemplated several times of rolling up sleeves to do it. I am glad it has now been done!

We thank the reviewer for the enthusiasm and the constructive feedback.

I do have some minor comments that need to be addressed, mostly regarding terminology:

1. The concept of canalization has historically been really poorly defined and as such causes many disputes among research groups. I suggest you make sure to define canalization as well as possible, especially with respect to using polymorphic lines for this study. For example, there has been some excellent work coming from Mark Siegal's lab showing that most of the genetic mutational buffering i.e. canalization is nothing but genetic background effect (eg https://doi.org/10.1371/journal.pbio.0060264 or https://doi.org/10.1371/journal.pgen.1003733), but this all depends on how you define robustness to mutation, and where the mutations are. If genetic canalization is being invoked it may be good to at least discuss the effects of variation at a single locus on different genetic backgrounds vs genetic variation at a locus while the rest of the genetic background is the same across lines. Similarly, when referring to canalization, please note if you mean genetic or environmental decanalization throughout the manuscript to avoid confusion.

We thank the reviewer for the suggestions. We have made several revisions to address this point.

1) We add an illustrative diagram in Figure 2 to depict what we mean by genetic

canalization/decanalization.

2) We add an illustrative diagram in Figure 3 to help understand the equivalency between several perspectives of describing canalization/decanalization and GxE.
3) We add a few sentences in the introduction to distinguish between robustness at the levels of genetic variation or environmental variation (L55-60), an important distinction to help understand genetic and environmental canalization/decanalization. The two references from the Siegal lab are perfect because one identified 300 capacitor gene for environmental variation and the other tested one of them for the ability to buffer genetic effects by new mutations. Thank you!
4) We adopt more consistent terminologies, adding qualifiers and explanations whenever confusion is possible.

We believe the clarity of the paper has been improved.

2. I think that often when referring to genetic variance you write "genetic variation" which may be confusing because when one hears "genetic variation" DNA variation across lines is the first thing that comes to mind and not the variation in gene expression across lines. This may be confusing.

We now use "genetic variance" to refer to between-line genetic variation.

L55: I think that because G x E is such a common abbreviation in the field using it over GEI may make the study more readable.

We change GEI to GxE throughout the text.

L21,57(and throughout the manuscript) : The term "plastic genetic architecture" does not sound right to me. Usually in the field plasticity refers to the phenotype and not to genetic architecture; it is not the genotype that is changing but more that the expression of the genotype is variable across environments. I see why in the case of GxE you could maybe call it plasticity of the genetic architecture, but this kind of nomenclature adds another layer of complexity on an already complex concept. It's kind of like genetic plasticity of plasticity; genetic variation or genetic variability would be better suited.

To avoid confusion, we have changed the term plastic genetic architecture to environmentally responsive genetic architecture throughout the text. The title has also been changed to adopt a more consistent terminology.

L19 (and throughout the manuscript): Because GxE refers to not just temperature but different rearing batches please change to "when flies developed at different environments". I think it was a fair point that you have highlighted this in L110, but I would also suggest to still be more careful at wording when declaring causative factors for change in variance. For example, in cases like when you find genetic decanalization at 18C and canalization at 25C, which may seem to starkly contradict the results by Chen et al. 2015, this difference may simply be a consequence of variance increasing due to other, unknown factor in this batch.

We completely agree and this has been changed.

L310 How do you reconcile this result with recent report of highly polygenic architecture in adaptation to novel thermal environments by Barghi et al. 2019?

We thank the reviewer for this very thought-provoking question. One connection between the robustness of co-expression network and polygenicity of organismal traits or fitness is that robust network can promote polygenicity. It is robust hence buffers the effects of mutation; it is networked hence the involvement of many genes. These can lead to many genes with small effects for a certain trait or fitness. We added some discussion of this point (352-357).

L148 For a reader used to the concept of canalization and plasticity this is a good walk-through interpreting or disentangling one from another. However, I believe a visual representation of these processes may substantially help the naïve reader and help you make your point! Maybe consider adding a schematic figure depicting the different concepts, perhaps even in the main text. For me, it was also sometimes difficult to follow which variance is being considered and in which context, so in case you opt out of a schematic figure, it would benefit the manuscript to try to reword this paragraph with a naïve reader in mind.

We thank the reviewer for the suggestion. This paragraph has been substantially revised to improve clarity. A few illustrative diagrams have been added to Figure 3 to depict the different perspectives of viewing GxE.

L163 change to "genotype by environment interaction"

Done.

Fig 2

In the caption point out that the significant points are blue, as well as red points.

Done.

Is there a particular reason why LOESS is used?

No. The relationship wasn't expected (and it wasn't) to be linear so we used LOESS as a smoothing method to highlight the trend. Any other smoothing method should work in this case.

Fig4

a) This is a very confusing figure, needs more explanation in caption at least, for example I was totally lost what the different numbers are and how they refer to the text where Fig 3 was referenced.

We thank the reviewer for the opportunity to clarify. We have revised the text and added additional explanation to the caption.

g) why permute over the whole set instead of within 25 C only?

We need to account for the fact that 25 °C and 18 °C share some eQTLs. We randomly sampled a set of SNPs and assign the same number of "eQTLs" to each category of 1) 25 °C only, 2) 18 °C only, and 3) both to preserve the sharing.

We did test randomly sampling within the 25 °C only set (keeping the other sets constant) and obtain highly similar results. Therefore we kept the original analysis as described.

Fig 6

I have to say that the correlation plots although significant, seem unconvincingly flat. Is the slight positive slope driven by the cluster of points with very low stabilizing selection values?

We did not expect the magnitude of correlation to be high given the complexity of the true model that we cannot even specify (may or may not be linear or monotonic). To be honest, we were quite happy to detect any signal at all. Apart from uncertainties in these estimates, the signal could have been diminished by the following factors, among others:

1) The strength of selection Vm/Vg is based on Vm and Vg estimated at 25 °C, this may not capture the true stabilizing selection because it is based on only one environment.
2) Gene connectivity is estimated by averaging absolute correlation across all genes. Depending on the network structure, e.g. many smaller modules with strong connectivity versus few large modules with small connectivity, this may not be the best proxy for the essentiality of a gene within the network.

Reviewer #1 (Remarks to the Author):

I was satisfied with this revision.

My only comment is a general one about the paper. Phrasing in some places seems overly wordy. I can't recall if I noticed this before. The title is a great example though: Response of Regulatory Genetic Variation in Gene Expression to Environmental Change in Drosophila melanogaster. I recommend the authors try to simplify the title and, where possible, the text. Doing so might help attract a broader readership.

I do not feel I need to review another version of this manuscript.

Reviewer #2 (Remarks to the Author):

The authors have satisfactorily addressed all reviewers' comments including mine. Specifically, some of the confusing terminology are redefined, and clear definition are provided. Providing schematics for these terminology is also a great help (Fig. 2e, 3a-c). I find the article has improved and it reads more nicely.

## Response to reviewers

Reviewer #1 (Remarks to the Author):

I was satisfied with this revision.

My only comment is a general one about the paper. Phrasing in some places seems overly wordy. I can't recall if I noticed this before. The title is a great example though: Response of Regulatory Genetic Variation in Gene Expression to Environmental Change in Drosophila melanogaster. I recommend the authors try to simplify the title and, where possible, the text. Doing so might help attract a broader readership.

I do not feel I need to review another version of this manuscript.

We thank the reviewer for the approval; the revision was greatly improved thanks to suggestions made by the reviewers. We have shortened the title to "Genotype by environment interaction for gene expression in *Drosophila melanogaster*". We also went through the text to simplify at various places to improve clarity.

Reviewer #2 (Remarks to the Author):

The authors have satisfactorily addressed all reviewers' comments including mine. Specifically, some of the confusing terminology are redefined, and clear definition are provided. Providing schematics for these terminology is also a great help (Fig. 2e, 3a-c). I find the article has improved and it reads more nicely.

We thank the reviewer for the approval; the revision was greatly improved thanks to suggestions made by the reviewers.
